# Chronic pain in Alzheimer disease and the role of neuroimmune interactions

**DOI:** 10.1016/j.pharmr.2026.100149

**Published:** 2026-06-26

**Authors:** Marzia Malcangio, George Sideris-Lampretsas

**Affiliations:** 1 Wolfson Sensory, Pain and Regeneration Centre, King’s College London, London, United Kingdom; 2 Department of Neuroscience, Physiology and Pharmacology, University College London, London, United Kingdom

## Abstract

Chronic pain conditions affect a large proportion of the global population and pain has tremendous personal and economic impact, affecting quality of life and ability to work while imposing a significant burden on families and health care systems. Neurological disorders and painful conditions are frequently accompanied by chronic pain, which is often jointly diagnosed with age-related neurodegenerative diseases, however, many patients do not receive adequate treatment. This review aims to create a working framework around the issue of under-recognized/undertreated chronic pain in Alzheimer disease by offering an in-depth overview of the mechanisms driving chronic pain and how we can leverage this knowledge to advance therapeutics approaches for these patients with an emphasis on the immune system.

**Significance Statement:**

Chronic pain conditions are prevalent among the aging population, and they positively associate with dementia. The incidence of chronic pain is associated with neuropsychiatric symptoms of dementia and pain management should aim for effective treatments regardless of cognitive status.

## Introduction

Pain is an unpleasant sensory and emotional experience in response to an actual or potential threat to the integrity of the body.^1^ Loss of this protective mechanism in individuals with congenital incapacity to perceive pain has considerable physical consequence and affects quality of life (QOL).^2^^,^^3^ Similarly, in people with neurodegenerative diseases, cognitive impairment may result in loss of capacity to report pain and poorly managed pain, which would have negative effects on their QOL.^4^

The acute sensation of pain, which we experience for instance after touching a hot surface, results from perception of such a feeling as painful. Acute pain disappears when the underlying cause is healed.^1^ The neuronal pathways that process pain start in peripheral tissues such as the skin, where noxious stimuli are detected and converted in sensory information by specialized neurons, which convey encoded stimulus information to the spinal cord on its way to the brain where pain is perceived. In some circumstances pain can last long after the initiating cause has healed and persists for months or even years. In the clinic, pain lasting more than 3 months from the initial cause is defined as chronic pain, a condition that exerts a debilitating impact on individuals’ QOL. Unlike acute pain, chronic pain is a disease on its own right, which is caused by ongoing changes in the pain pathways and associated with symptoms such as increased sensitivity to painful stimuli (hyperalgesia) and a painful response to previously nonpainful stimuli (allodynia), alterations of mood, cognitive function, family and social relations, and sleep health.^5^, ^6^, ^7^

Clinical classification of chronic pain includes neuropathic, nociceptive, and nociplastic pain, with mechanistic changes in peripheral nervous system (PNS) and central nervous system (CNS) leading to enhancement and persistence of pain. The neuroanatomical routes for pain signaling include both ascending inputs from peripheral sensory neurons to projection neurons in the spinal cord on their way to the higher centers in the brain where pain is perceived, and descending circuits from the brain to the spinal cord that modulate the experience of pain (Fig. 1). Neuropathic pain is caused by a damage to the nervous system, which leads to ongoing or intermittent pain as well as pain evoked by light touch and innocuous cold (allodynia).^8^ Nociceptive pain after tissue damage or infection is associated with activation of the immune system and release of inflammatory mediators that increase sensory sensitivity. Although inflammatory processes aim to promote healing and limit the extent of damage, this adaptive response is hijacked in diseases such as rheumatoid arthritis and results in the establishment of chronic pain with persistent hyperalgesia and allodynia.^9^ Finally, nociplastic pain features wide-spread pain, amplification of pain processing and/or decreased inhibition at multiple levels in the nervous system without objective evidence of ongoing inflammation or nerve damage, such as in bladder pain syndrome and fibromyalgia.^10^, ^11^, ^12^

**Fig. 1:**
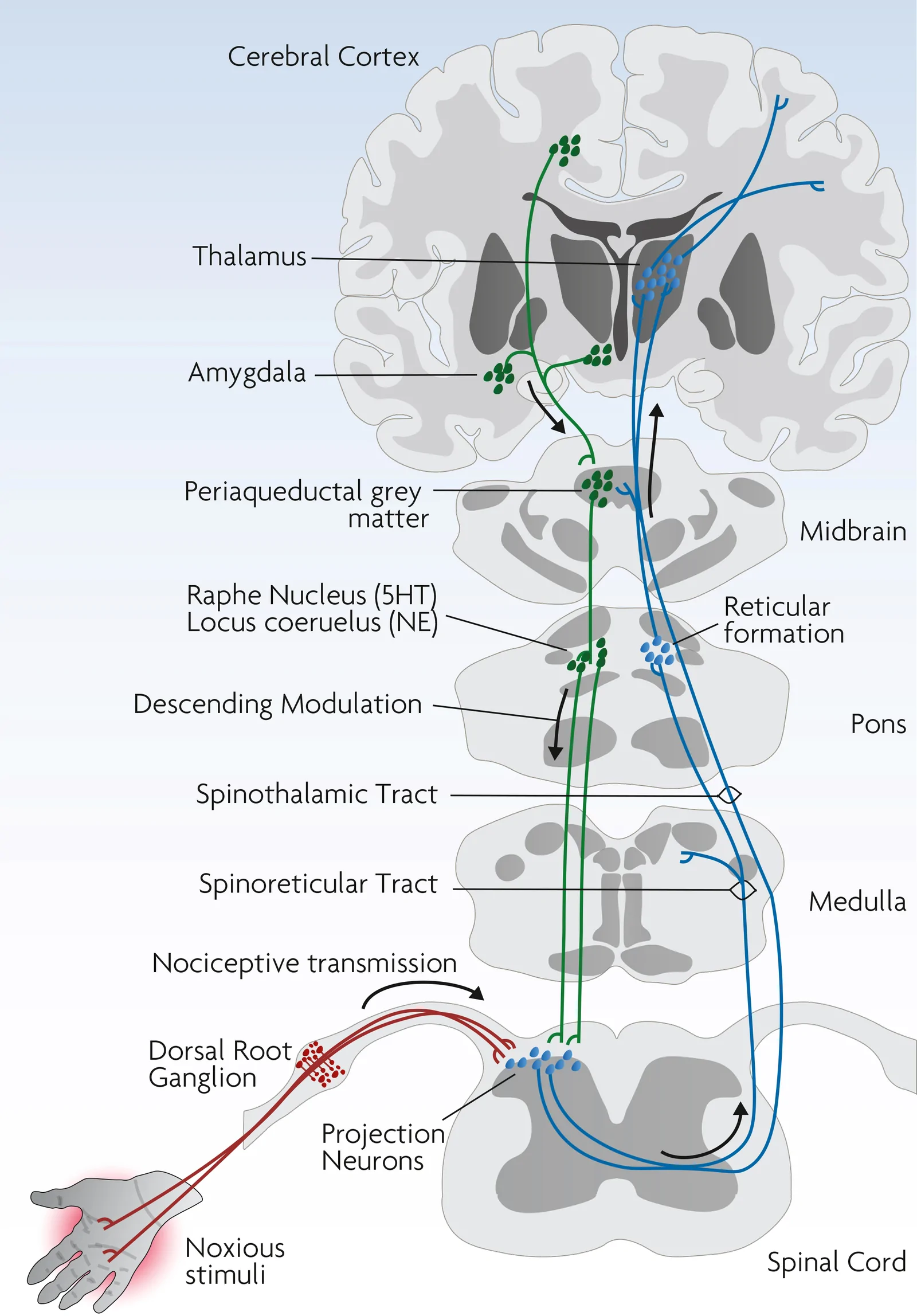
Ascending and descending pain pathways. Noxious stimuli (thermal, mechanical, and chemical) are detected in peripheral tissue (hand) by specialized neurons with cell bodies in the DRG that send branches, which enter the spinal cord where they synapse in the gray matter of the dorsal horn. Axons of the second order neurons cross the midline and ascend to the brainstem and thalamus in the contralateral quadrant of the spinal cord (blue pathways). Third order neurons ascend to the cortex where pain is perceived. Descending pathways (green) are controlled by interactions with brain centers and relay information from higher centers back to the spinal cord where they modulate the processing of sensory information. Within the spinal cord are axons descending from the periaqueductal gray (PAG) matter (shown in green) and from the rostral ventromedial medulla (RVM) and the hypothalamic A11 dopaminergic neurons (not shown), which normally result in the inhibition of the activity of nociceptive projection neurons.

Chronic pain is the primary reason for people to seek medical care and constitutes a major cause of persistent disability. In the United Kingdom, more than 40% of the population, meaning around 28 million adults, are living with chronic pain.^13^ Chronic pain is often jointly diagnosed in age-related neurodegenerative diseases, the main preliminary cause for dementia.^14^ The umbrella term “dementia” describes memory loss, cognitive impairment and progressive inability to perform daily tasks. Alzheimer disease (AD) accounts for 60%–80% of dementia cases with the remaining 20% attributed to vascular dementia, a group of disorders characterized by reduced cerebral blood flow and overt cerebrovascular pathology. Moreover, chronic pain conditions occur in concomitance with neuropsychiatric symptoms of dementia such as anxiety, aggression, and depression.^15^ Although pain is not a primary symptom of dementia, more than half of people with dementia daily experience pain, and painful conditions such as musculoskeletal disorders affect majority of patients, including people with AD.^16^^,^^17^ In addition to direct distress, pain may further impair function, exacerbate depression, and other mood disorders and increase agitation and irritability, all impacting on QOL.^15^ Cognitive impairment is a risk factor for the undertreatment of pain due, at least in part, to individuals’ progressing inability to communicate.^18^ People with cognitive impairment either receive less prescription of analgesics compared with cognitively intact individuals^19^ or receive inappropriate treatment.^15^ Furthermore, poor treatment of pain results from a lack of understanding of the impact of neurodegenerative pathology on factors that influence the experience of pain.^14^ Thus, considering that the risk of untreated/undertreated pain increases with severity of dementia and pain compromises QOL, there is an unmet need for proper management of pain, aiming for effective pharmacological treatment regardless of cognitive status.

This review aims to create a working framework around the clinical issue of under-recognized/undertreated chronic pain in people with dementia with a focus on people with AD. We first discuss the clinical challenges associated with pain assessment and altered pain perception in people with dementia including people with AD. Moreover, we report clinical evidence that AD pathology features neuronal changes and immune cell activation in brain areas involved in pain processing. Then, with the aim to delineate possible mechanisms of chronic pain in AD, we examine preclinical evidence suggesting that neuroimmune mechanisms involving microglia and astrocytes contribute to nociceptive processing in models of chronic pain and in transgenic mouse models of AD. Finally, we highlight potential therapeutic targets for pain in AD emerging from neuroimmune shared mechanisms.

## II Dementia and chronic pain: Focus on Alzheimer disease

Chronic pain conditions are prevalent among the aging population and stem from musculoskeletal and neuropathic conditions.^20^ In the elderly, persistent pain is associated with cognitive decline and a higher risk of dementia.^21^ However, mechanisms of chronic pain remain largely unexplored. Based on existing evidence that pain perception is altered in people with dementia including people with AD, we examine the possibility that AD pathology involves discrete and specific mechanisms that could result in changes in pain signaling and alteration of pain sensation and perception. Despite aging being the most significant risk factor for AD, brain changes that characterize AD neuropathology start decades before the manifestation of cognitive symptoms.^22^ The AD brain features extracellular senile plaques and intraneuronal neurofibrillary tangles (NFTs): senile plaques result from deposition of *β*-amyloid (Aβ) peptides derived from neuronal amyloid precursor protein (APP) cleavage by presenilin-1 (PSEN1) and presenilin-2 (PSEN2); and NFTs result from intracellular hyperphosphorylation of tau protein that normally maintains microtubule stabilization.^23^ Amyloid plaques and tau filaments are neurotoxic and associated with astrogliosis,^24^ microglial activation, which can also be neurotoxic,^25^^,^^26^ and with prominent decrease in cerebral blood flow due to capillary constriction (Fig. 2).^27^ AD pathology follows a specific temporal and spatial pattern in the brain, and, critically, tau and amyloid pathology show distinct patterns of manifestation and progression.^28^, ^29^, ^30^ Recent genetic, molecular and omics approaches suggest that glial and immune cells contribute to AD pathogenesis and progression.^31^ Indeed, astrocytes and microglia undergo morphological, molecular, transcriptional, and functional changes during the decade-long preclinical stage of AD.^32^

**Fig. 2:**
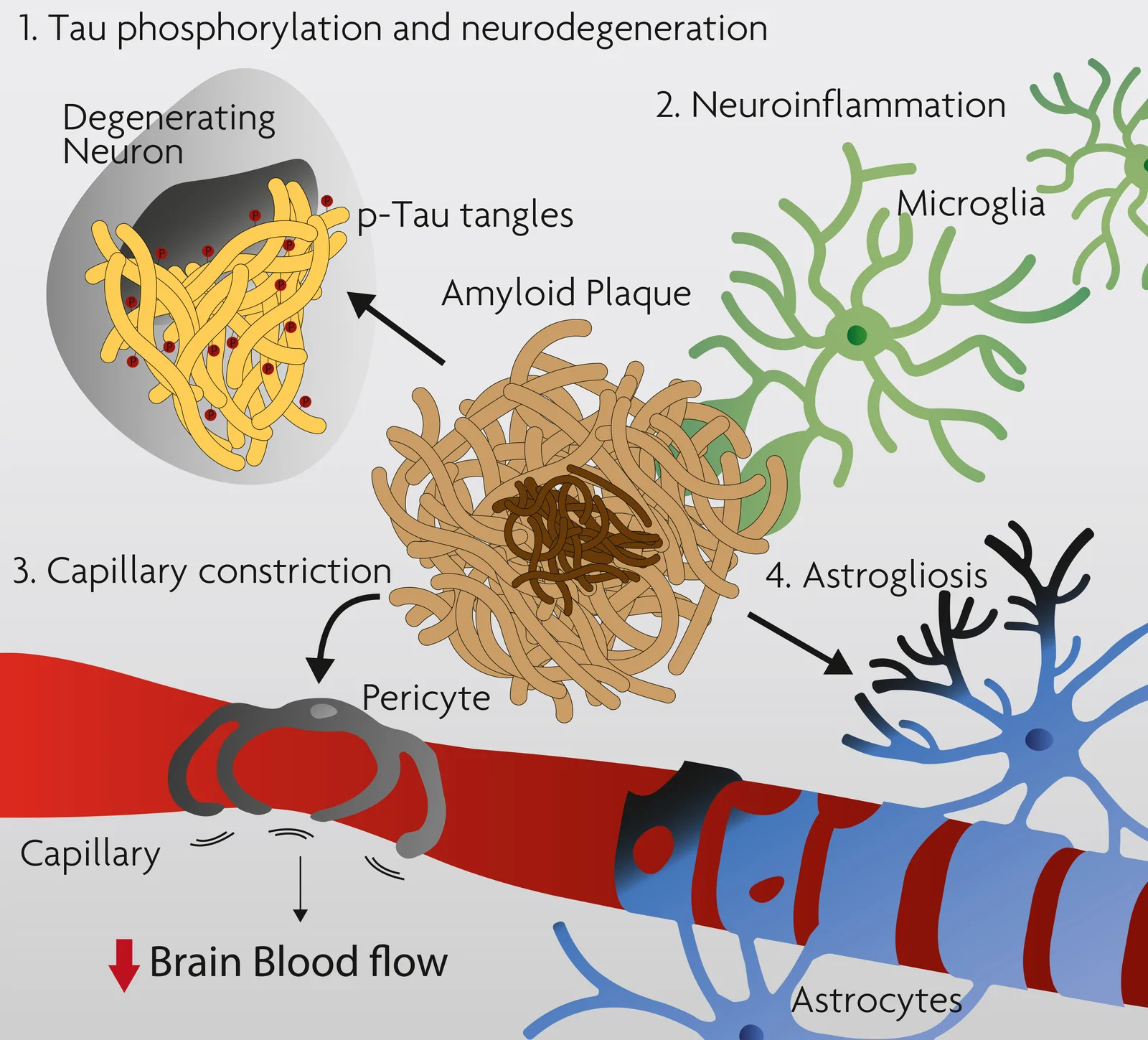
The neuropathology of Alzheimer disease. The major pathological hallmarks of AD include parenchymal amyloid deposition (brown) and intraneuronal tau hyperphosphorylation and subsequent aggregation (1 – yellow). Tau aggregation leads to the formation of neurofilament tangles that are closely associated with neurodegeneration in AD. These pathological hallmarks lead to loss of synapses and synaptic plasticity, which result in cognitive impairment. In addition, amyloid plaques and tau tangles induce a strong response in the tissue resident immune cells, namely microglia (2 – green). Genome-wide association studies position microglia at the epicenter of AD pathogenesis, as several risk factor genes are enriched in microglia and affect their ability to respond to amyloid deposition. In addition, elevated levels of oligomeric amyloid early in the disease process induce potent capillary constriction by promoting pericyte contraction (3 – gray) that results in a significant reduction of cerebral blood flow, which is the first clinically detectable symptom of AD. Finally, accumulation of amyloid is linked to pronounced astrocyte reactivity (4 – blue), leading to astrocytic end feet retraction and their inability to maintain synaptic homeostasis and vascular health.

Besides aging, other AD risk factors include cardiovascular diseases, lifestyle, and gender as females are more likely to develop AD, especially after the age of 80 years.^33^ Genetic risk factors include rare dominantly inherited mutations in *APP*, *PSEN1*, and *PSEN2* that lead to early-onset familial AD, which accounts for less than 5% of AD cases with an age of onset as early as the third decade of life. Sporadic AD, which makes up most of the cases, is diagnosed in individuals over the age of 65 years. Genetic risk factors for sporadic AD include variant in *APOE ε*4 gene encoding apolipoprotein and variant in the encoding gene for the triggering receptor expressed on myeloid protein 2, *TREM2* variants in microglia.^34^ Relevant to the topic of this review, in human and mouse spinal cord microglia polymorphisms in *APOE* are also associated with chronic pain conditions.^35^ Furthermore, preclinical evidence demonstrates microglial activation in brain and spinal cord in transgenic mouse models of AD under concomitant nociceptive pain conditions such as rheumatoid arthritis and neuropathic pain.^36^ In this article, we discuss a potential interaction between the pathophysiology of chronic pain and AD, with microglial activation. Such a mechanism could serve as a potential starting point for understanding this complex interplay and for developing novel immunity-oriented therapeutic approaches that combat both neurodegeneration and jointly diagnosed chronic pain.^37^ In addition, we discuss that growing evidence describes a role for astroglia in chronic pain and a prominent role of astrogliosis in AD.^38^^,^^39^ However, we note that whether AD reactive astrocytes play a mechanistic role in pain in AD remains to be explored.

## III Pain prevalence, pain assessment, and pain identification in people with dementia including people with Alzheimer disease

Considering that aging is a risk factor for both chronic pain and AD, the higher prevalence of acute and chronic pain in older people constitutes a significant public health concern.^20^ Indeed, a proportion of individuals diagnosed with AD are also diagnosed with chronic pain.^40^, ^41^, ^42^ Despite remarkable effort at developing instruments to identify and measure pain, the assessment of pain intensity (pain thresholds), pain tolerance (endurance), and pain prevalence remains challenging in individuals living with dementia, including people with AD. For instance, the diversity of clinical manifestations of AD combined with progressive deterioration of cognitive and communication abilities complicates pain assessment.^43^^,^^44^ Furthermore, studies in AD, dementia cohorts, and institutionalized older populations are often mixed together and this results in variable outcomes (Table 1).^43^, ^44^, ^45^, ^46^, ^47^, ^48^, ^49^

**Table 1:** Prevalence of pain in dementia including dementia in people with AD

Study	Pain Prevalence	Pain Assessment	Care Setting	Stage of Dementia
Ferrell et al^43^	• 62% reported pain complains during the interview and pain questionnaire	Cross-sectional interview McGill Pain Questionnaire	Skilled nursing homes	MMSE 12.1 ± 7.9 Severe cognitive impairment
Malara et al^44^	• NRS 42% • PAINAD 51.8%	Self-reporting NRS and observational tools PAINAD	Nursing homes	DSM IV; MMSE moderate-severe
Van Kooten et al^45^	• 43% MOBID-2 • 26.9% PAINAD • Self-reported pain residents with vascular dementia (54%) with Alzheimer disease (18%) and other dementia subtypes (14%)	Observational MOBID-2 pain scale and self-report	Nursing homes	GDS 6 Moderately severe stage of dementia
Sampson et al^46^	• Self-report: 27-39% • PAINAD: 19% at rest; 51% on movement	Self-report; PAINAD	General hospital	MMSE ≤ 24
Shega et al^47^	• Self-report 32% • 52% of caregivers report pain in their care recipients • 59% of dyads agree on the presence of pain	Self-report and VDS	Community-dwelling persons with dementia and their family caregiver	MMSE ≤ 10
Hendriks et al^48^	• 47%–68% observational	Observational	Nursing homes	GDS 7 CPS 5, 6
Rajkumar et al^49^	• 35.3%; APS score 3–7	APS observational assessment	Care homes	FAST: mild to moderately severe dementia

APS, Abbey Pain Scale; CPS, Cognitive Performance Scale; DSM IV, Diagnostic and Statistical Manual of Mental Disorders, Revised Fourth Edition; FAST, functional assessment staging tool; GDS, Global Deterioration Scale; MMSE, mini mental state examination; NRS, Numeric Rating Scale; PAINAD, Pain Assessment in Advanced Dementia scale; VDS, Verbal Descriptor Scale.

In clinical research settings, the approaches that are adopted to measure the integrity of pain processing include brain imaging, measurements of somatosensory evoked potentials for assessing the function of the sensory pathway, nociception flexion reflex for assessing spinal excitability, as well as measurements of pain thresholds and pain tolerance.^50^ However, in the more common clinical practice, assessments of pain in cases of mild to moderate dementia relies on self-reporting, which is often associated with observational tools such as interview-based assessment scales. Specifically, the Pain Assessment in Advanced Dementia (PAINAD) scale is a 5-item observational tool developed to assess pain in people with severe dementia. The items include breathing, independent vocalization (1–6 definitions), negative vocalization (1–6 definitions), facial expression (1–5 definitions), body language (1–9 definitions), and consolability (1–3 definitions). Each item is scored 0–2 and a total score of 2 or more can be used as an indicator of probable pain.^51^^,^^52^ The Mobilization-Observation-Behavior-Intensity-Dementia-2 (MOBID-2) Pain Scale is an observational tool to assess pain behavior in people with moderate to severe dementia. Pain assessment is based on pain behavior related to the musculoskeletal system in relation to guided movements during morning care (MOBID-2, part 1, 5 items). Evaluation of pain intensity is based on vocalization, facial expression, and defense (part 1). MOBID-2 part 2 assesses behavior related to internal organs, head and skin. A total MOBID-2 is based on a total of 10-point: no or mild pain (range 0–2), moderate pain (range 3–6), and severe pain (range 7–10).^53^ Finally, the Abbey Pain Scale is a 1-minute numerical indicator for people with end- or late-stage dementia who are unable to articulate their needs. Caregivers score 6 observation categories including vocalization, facial expression, change in body language, behavioral change, physiological change, and physical change. Each category is scored from 0 (absent) to 3 (severe) and total scores of 14+ indicates severe pain.^54^

We reason that it is quite possible that pain assessment in community settings, which often relies on self-reporting, may be compromised by limited understanding among patients and caregivers, as well as by less consistent monitoring routine compared with clinically trained staff in nursing homes. Such logic would also apply to the variability of pain prevalence in people with dementia including people with AD, which ranges from 20% to 70% of the patients screened (Table 1). Other reasons for such a large variability are manifold and introduced by grouping all people living with dementia in one group rather than stratifying them based on the specific underlying pathology or stage of disease.^45^ In addition, the different outcomes between self-reporting and either observational scales such as PAINAD, Abbey Pain Scale, and MOBID-2^43^^,^^44^^,^^46^ or verbal descriptor scales^47^ contribute to variability. Different outcomes are evident when comparing pain at rest and pain on movement,^46^ self-report and reports by patient-caregiver dyads,^47^ observational assessment reports in either nursing homes or care homes.^48^^,^^49^ Notably, observed pain (PAINAD scale) is associated with psychiatric symptoms of dementia (Behavioral Pathology in Alzheimer Disease Scale) such as aggression and anxiety^46^ suggesting that poor pain management may trigger distress and aggressiveness.^15^

According to the seminal Einstein Aging Study, one of the longest-running prospective cohort studies of aging, the degree of pain-related impairment in activities of daily living, termed pain interference, is associated with a higher probability of developing dementia, although there is no significant correlation between intensity of pain and development of dementia.^55^^,^^56^ Notably, a study investigating unplanned hospital admissions in people living with dementia indicates that more than 10% of such people experiencing pain received no analgesics during their admission.^46^ This is alarming as it indicates a caveat in the diagnosis of chronic pain in individuals with AD and encourages implementation of improved standard operating procedures when working with older individuals that may or may not have received a dementia diagnosis. In addition, presence of chronic pain is associated with higher risk of incident (newly diagnosed cases) AD and related dementias,^57^^,^^58^ suggesting that pain assessment should become an integral part of dementia diagnosis. Nevertheless, it is undeniable that proper assessment of pain in people with AD remains difficult with pain either overestimated or underestimated, making it difficult to pinpoint the real situation.

In conclusion, the shared incidence of chronic pain and AD requires clinical guidance and validated assessment tools to successfully detect and manage pain in people with dementia.^15^^,^^40^, ^41^, ^42^ The inability to report chronic pain is a good predictor of accelerated cognitive decline and it is positively linked to behavioral symptoms such as agitation, aggression, and depression.^46^^,^^48^ Importantly, chronic pain is associated with an all-cause mortality in follow up studies,^49^ suggesting that its impact on patient frailty and overall QOL is disproportional to the scope, depth and comprehensiveness of published research on pain.

## IV Pain processing in Alzheimer disease

The complex nature of the pain experience in AD is exemplified by evidence that people with AD can detect painful stimuli and distinguish them from non-noxious touch,^59^ although both intensity of pain (sensory-discriminative) and pain affective component (emotional-affective) are altered. Thus, according to some experimental pain reports, pain thresholds can be higher than in cognitively intact individuals,^14^^,^^59^, ^60^, ^61^ but self-reported pain unpleasantness ratings can be either comparable to controls or increased.^14^^,^^62^

Compared with healthy controls, people with AD require higher temperatures to report sensing mild and moderate pain, indicating that they are less sensitive to thermal pain.^14^^,^^62^ However, other evidence indicates that, in people with AD, detection of an electrical stimulus to the wrist appears comparable to cognitively intact individuals.^59^ Self-reported unpleasantness ratings of mild and moderate thermal pain do not appear to differ from controls, indicating that in people with AD, pain does not become less unpleasant.^14^ Nevertheless, when facial coding is used as an alternative to verbal reports of pain to measure affective responses to evoked pain, facial responses to mechanical pressure and electric stimuli are exaggerated.^63^, ^64^, ^65^ After application of noxious pressure, neuroimaging evidence indicates an enhanced connectivity in AD brain including areas such as the dorsolateral prefrontal cortex, anterior midcingulate, periaqueductal gray, thalamus, and hypothalamus.^66^ In such studies, affective modulation and altered pain emotional-affective components are inferred from the activation of brain regions involvement while unpleasantness is not measured.

Overall, when compared with healthy individuals, people with AD seem to have altered detection levels for painful stimuli but unpleasantness of pain appears either not changed or increased. However, this evidence is inconsistent between studies probably because of several factors including: (1) different modalities used to evoke pain such as electrical stimulation and arm ischemia versus thermal stimulation using a probe applied to the skin; (2) diverse outcome measurements, such as heart rate (autonomic pain response), electroencephalogram, and verbal reports versus quantitative sensory testing, assessment of warmth and heat pain thresholds, contact heat- and somatosensory evoked potentials; and (3) different stages of disease progression.^59^^,^^62^^,^^67^

Less variable data exist for pain tolerance, which appears higher in people with AD since perception of a strong painful stimulus (measured by numerical rating scale) is blunted even though the autonomic system response (heart rate) is comparable to non-AD people. Indeed, people with AD manifest increased tolerance to ischemic pain and noxious electrical, but not pressure stimulation.^59^^,^^62^ Thus, the magnitude of stimulus intensity required for the stimulus to be reported as unbearable is higher in AD.

Interestingly, pain tolerance increases with progression of cognitive impairment as assessed by mini mental state examination and electroencephalogram, indicating an alteration in pain tolerance that depends on cognitive and emotional-affective factors.^59^ Furthermore, cognitive impairment, as indicated by lower mini mental state examination scores between mild and severe AD, correlates positively with fewer changes in autonomic response to pain (heart rate increases after pain stimulation).^68^ In patients with AD who have mild or severe cognitive impairment, a reduced autonomic response to pain applies also to response to verbal warning that noxious stimuli are going to be delivered. The more severe the cognitive impairment the smaller is the anticipatory increase of heart rate.^68^ However, one needs to consider that when autonomic function is used as the outcome measure in people with AD, the development of psychiatric symptoms such as anxiety may be a confounding factor.

In imaging studies in people with AD, evidence indicates that an increase of neural activity in the pain pathway is accompanied by greater activity in cognitive processing regions suggesting that the cognitive integration of the painful experience may be altered.^61^ Furthermore, placebo analgesia is profoundly reduced in AD, possibly because of reduced frontal lobe connectivity (electroencephalogram analysis) with the rest of the brain.^61^^,^^69^ Placebo analgesia refers to the additive effect of expectation of therapeutic benefits/placebo-related mechanisms and drug-induced analgesia. In other words, analgesics are less effective if the placebo effect is absent. In people with AD there is disruption of placebo(expectation)-related analgesic mechanisms, which results in reduced efficacy of analgesics.

Overall, there is substantial evidence that tolerance to painful stimuli is altered in AD and loss of endogenous expectation and placebo mechanisms makes analgesic treatments less effective. Nonetheless, it is clear more studies are required to delineate changes in pain detection (sensory-discriminative component) of pain. We suggest that there is an urgent need for more assessment tools and tests^15^^,^^21^ together with their validation through longitudinal studies using neuroimaging, behavioral and psychophysical approaches. Ideally, these analyses ought to be complemented with quantification of biomarkers and should be considered in relation to disease stage and severity.

## V Mechanisms of chronic pain versus acute pain

A better understanding of how AD alters pain processing requires consideration of mechanisms underlying chronic pain states, which have been mostly generated by research in preclinical models in rodents. Here, we briefly describe the basic physiology of pain mechanisms and changes that are responsible for acute pain turning to chronic pain with particular emphasis to mechanisms involving neuroimmune and neuroglial signaling pathways. Thus, painful stimuli are detected by specialized sensory neurons, namely the nociceptors, with free nerve endings that are widely located in the skin, muscle, joint capsule, bone, and viscera. Nociceptors are pseudounipolar neurons with small cell bodies in the dorsal root ganglia (DRG) and a single axon that bifurcates to project one afferent to peripheral tissues and another afferent to the spinal cord.^70^ Nociceptive afferents, including A*δ* and C fibers, are thinly myelinated/unmyelinated, slow conducting and transmit noxious stimuli. They contain neuropeptides such as calcitonin-gene-related peptide and substance P. Instead, non-noxious, proprioceptive stimuli are transmitted by A*β* fibers that are myelinated, fast conducting and with large cell bodies in the DRG.^1^

In the dorsal horn of the spinal cord, primary afferents make synaptic contact with dorsal horn neurons including projection neurons and excitatory/inhibitory interneurons, some of the latter contain opioid peptides, namely enkephalins.^71^^,^^72^ Projection neurons ascend to brain areas via spinoparabrachial-limbic and spinothalamic-cortical pathways, which mediate emotional and sensory components to pain, respectively.^73^ Generally, supraspinal areas exert descending inhibition of nociceptive transmission at spinal cord level through raphe nucleus magnus-derived serotonergic pathway, noradrenergic pathway stemming from the locus coeruleus (LC) and adjacent pontine regions^74^ and the A11 dopaminergic system located in the posterior hypothalamus.^75^ However, the rostroventral medulla (RVM) descending modulation of pain includes OFF-cells and ON-cells, which exert inhibitory and facilitatory control over nociceptive transmission.^76^

In animal models of chronic pain after either peripheral nerve injury or tissue damage, which both feature behavioral allodynia and hyperalgesia, injured axons and fiber terminals are sensitized by inflammatory mediators produced locally by immune cells.^77^, ^78^, ^79^ Such a *peripheral sensitization* results from activation of ion channels and transduction channels^80^^,^^81^ and long-term changes in sensory neurons that lead to gene expression alteration and post-translational modifications within the DRG cell bodies.^82^, ^83^, ^84^ Overall, an increased peripheral input to the spinal cord, leads to *central sensitization*, which represents a state of hyper-responsiveness of dorsal horn neurons^85^^,^^86^ and it is orchestrated by several mechanisms including abnormalities in descending pathway control of sensory events^87^ and immune cell- and glial cell-mediated facilitation of nociceptive processes involving the microglia and astrocytes.^38^^,^^88^ Being resident immune cells, microglia constantly survey the parenchyma via highly dynamic processes and interactions with neurons, vasculature, and other glial cells.^89^ Astrocytes influence neuronal activity, control local blood flow, and interact with other glial cells, including microglia.^90^ They have a stellate shape with large and finer processes that enwrap neurons and synapses, and characteristic endfeets that contact blood vessels. Both microglia and astrocytes are involved in many different CNS physio-pathological conditions. In preclinical models of neuropathic pain after peripheral damage, dorsal horn microglia respond to increased peripheral input and hyperactivity of dorsal horn neurons, and contribute to persistent noxious signaling from spinal cord neurons to pain-related areas in the brain.^91^, ^92^, ^93^ Dorsal horn astrocytes become hypertrophic and undergo molecular and functional changes associated with distinct state of activation and contribute to chronic pain mechanisms by facilitating noxious signaling.^38^^,^^90^^,^^94^ We have recently reviewed the role of microglia in the induction and maintenance of chronic pain mechanisms^36^ and below we briefly describe microglial pathways that offer pharmacological targets for innovative therapeutic approaches for chronic pain. The role of astrocytes in chronic pain has been reviewed by us and others^38^^,^^90^^,^^94^ and we briefly cover neuron–astrocyte–microglia interactions.

### A Neuron–microglia interactions in chronic pain

In animal models of chronic pain after peripheral damage, dorsal horn microglia proliferate and accumulate in vicinity of the central terminals of primary afferent fibers, which are damaged peripherally and convey an exaggerated barrage of information to the spinal cord. Microglia change their profile in response to the increased activity of the synapse between afferent fiber terminals and dorsal horn neurons. Thus, clusters of microglia upregulate transcripts that include *Ctss, Cx3cr1, Itgam, and Bdnf* in models of neuropathic pain and *Lgals3* and *P2y12* in models of inflammatory pain.^37^^,^^95^

Colony-stimulating factor 1 is released by injured fiber terminals and binds colony-stimulating factor 1 receptor/DAP12 on microglia inducing expression of pain-associated genes (Fig. 3).^96^ In addition, microglia respond to other factors released by primary afferent terminals, including ATP (which is also released by astrocytes) and galectin-3 (Gal-3). Such factors engage specific communication pathways with neurons and facilitate nociceptive signaling. For example, microglia express P2X4 and P2X7 receptors that interact with neuron- and astrocyte-derived ATP: this mechanism is validated by the antinociceptive effects of receptor antagonists in animal models of neuropathic pain. Microglia P2X4 mediates release of brain-derived neurotrophic factor that causes loss of GABA-mediated inhibitory tone and facilitates nociceptive mechanisms (Fig. 3).^97^ Microglial P2X7 promotes release of cysteine protease cathepsin S that liberates the chemokine domain of neuronal fractalkine (CX3CL1). Soluble CX3CL1 activates CX3CR1 on microglia and leads to the secretion of pronociceptive cytokine interleukin-1*β* (IL-1*β*) (Fig. 3).^98^, ^99^, ^100^ P2X7 antagonists, cathepsin S inhibitors and CX3CR1 antagonists exert antiallodynic effects in models of neuropathic pain.

**Fig. 3:**
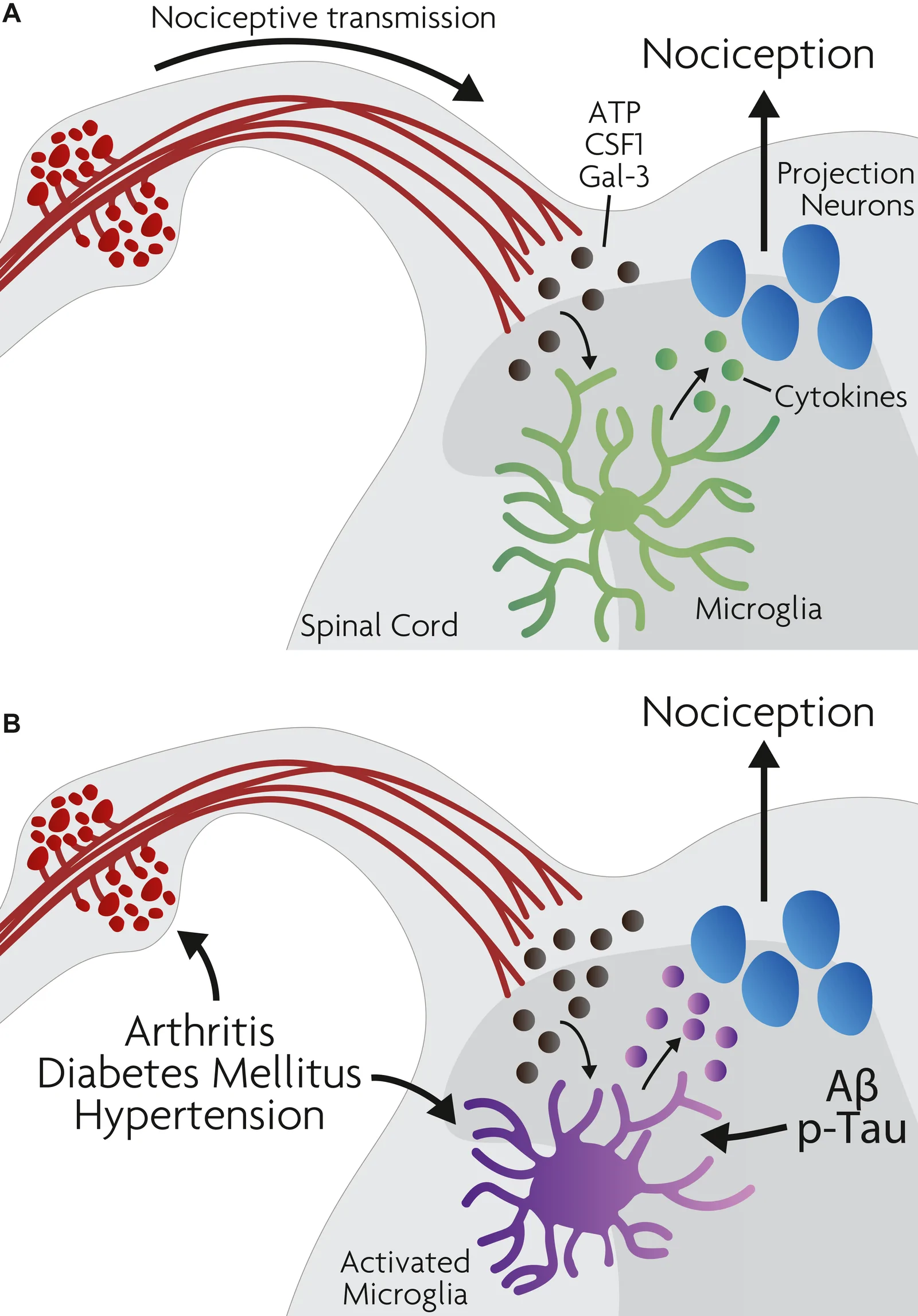
Alzheimer disease neuropathology and pain processing. (a) Preclinical evidence indicates that during neuropathic nociception, injured sensory neurons release ATP, colony-stimulating factor 1 (CSF1), Gal-3, and other mediators from their axon terminals in the dorsal horn of the spinal cord. These mediators exert pronociceptive functions through interactions with dorsal horn microglia. After their activation, microglia release a set of cytokines, such as IL-1*β*, which potentiate nociceptive transmission in projection neurons of the dorsal horn. (b) Common clinical conditions jointly diagnosed with AD such as diabetes mellitus, rheumatoid arthritis, and hypertension are routinely modeled in rodents. In such models, evidence indicates changes in the composition (CSF1) and quantity (Gal-3) of sensory neuron mediators released in the dorsal horn and activation of microglia. In addition, in mouse models of AD, elevated levels of soluble oligomeric amyloid and tau in the CSF directly “activate” microglia, altering their ability to respond to sensory neuron-derived mediators. It is the assertion of this review that targeting altered microglial properties in AD might offer a novel therapeutic approach to treating altered pain in AD.

Another pathway that is emerging from preclinical studies as a potential therapeutic target is the Gal-3/Toll-like receptor 4 (TLR4) pathway. Specifically, after nerve injury and tissue damage, some clusters of dorsal horn microglia upregulate TLR4, which mediates microglia response to Gal-3 released by primary afferent fibers (Fig. 3).^95^^,^^101^^,^^102^ TLR4 is a potential therapeutic target for pain,^103^ and targeting sensory neuron-derived Gal-3 might be beneficial against both neuropathic and inflammatory nociception.

Beside the dorsal horn of the spinal cord, alterations in microglia properties occur in supraspinal areas that are closely linked with both the sensory-discriminative and the affective-motivational component of chronic pain.^104^ Indeed, in animal models of neuropathic pain after nerve injury, microglia are activated in contralateral primary sensory cortex, thalamus, ventral tegmental area, nucleus accumbens, and amygdala.^105^

In addition, multiple preclinical reports in rodents link aberrant microglial functions with the development of depression-like behavior, and memory deficits.^106^^,^^107^ In particular, several weeks after the development of neuropathic allodynia, the detection of depression-like behavior is associated with microgliosis and soma enlargement in the hippocampus, amygdala, and medial prefrontal cortex.^106^^,^^107^ Relevantly, microglia become more phagocytic and release proinflammatory cytokines such as IL-1*β* in the nucleus accumbens, prefrontal cortex, and hippocampus, which is associated with memory deficits and depression-like behavior.^108^^,^^109^ The pronociceptive role of IL-1*β* is reinforced by observations that administration of an anti-IL-1*β* antibody or genetic deletion of IL-1*β* receptor (IL-1R1) can prevent microgliosis in the medial prefrontal cortex, hippocampus, amygdala, and nucleus accumbens: such a morphological effect is matched by a reduction in depression-like behavior in rodents in neuropathic conditions.

It is relevant to remind, herein, the emergence of clinical evidence for microglial activation in chronic pain states. Thus, (micro)glial responsiveness is evident in human brain in people with chronic pain as demonstrated by positron emission tomography (PET)-imaging studies using the translocator protein tracer TSPO-[^11^C]-PBR28, which binds microglia and astrocytes. In people with low back pain glial cells are activated in both thalamus and paracentral lobule of the somatosensory cortex.^110^

### B Neuron–astrocytes–microglia interactions in chronic pain

In animal models of neuropathic pain after peripheral nerve injury, dorsal horn astrocytes respond to both heightened neuronal activity and microglial activation through transition into a reactive state characterized by an alteration in morphology, exemplified as hypertrophy, and increased expression of glial fibrillary acidic protein. Neurotransmitters such as glutamate and ATP induce release of intracellular calcium in astrocytes with the consequent propagation of calcium waves that stimulate adjacent astrocytes.^90^ Propagation of calcium waves and increase of intracellular calcium induce release of ATP and glutamate, which facilitate neuronal activity and nociceptive signaling. Consistently with a mechanistic role of astrocytes, block of calcium wave propagation reverses neuropathic allodynia.^90^ Reactive astrocytes are a major source of ATP that activates P2X3 receptor expressed by nociceptive fiber terminals, P2X4 and P2X7 in microglia and P2X7 in reactive astrocytes. Thus, ATP mediates astrocyte–astrocyte interactions and astrocyte communication with microglia and neurons. Astrocytes also express cytokines such as tumor necrosis factor-*α* (TNF-*α*) and IL-1*β* and chemokines such as chemokine C-C motif ligand 2 (CCL2), which is released by TNF-*α* application to astrocytes.^111^ CCL2 activates CCR2 receptor expressed by dorsal horn neurons and facilitates nociceptive signaling. Thus, such astrocyte–neuron–microglia interactions contribute to spinal cord mechanisms that underlie induction and maintenance neuropathic allodynia.

Moreover, reactive astrocytes can be observed in supraspinal regions such as the RVM that is major component of brainstem descending pain modulatory circuitry.^112^ RVM reactive astrocytes express IL-1*β* and TNF-*α* which activate TNFR1 and IL-1R expressed by RVM neurons and enhance descending facilitation of neuropathic pain.

Clinical evidence for involvement of astrocytes in people with chronic pain is sparse and clinical trials with putative astrocyte inhibitors have been unsuccessful.^113^ This outcome is not surprising when considering that human and rodent astrocytes show differences in size, number of processes, and number of neuronal domains. Nevertheless, there is clinical evidence of possible astrocyte association with pain, alongside microglia, which can be inferred from TSPO-PET in vivo imaging studies in people with back pain, fibromyalgia, and migraine.^114^ Indeed, in brain and spinal cord regions related to pain, TSPO tracer binding includes both astrocytes and microglia, which normally express low levels of TSPO that increases significantly when cells are activated.^114^ However, TSPO elevation in astrocytes appears of limited relevance in patients with fibromyalgia where signal elevation is mainly attributed to microglia.^115^ Lack of TSPO cell specificity remains an issue alongside the complexity of binding data acquisition and analysis.^114^^,^^116^ Despite having gained popularity to evaluate evidence of inflammation in the brain in various conditions, TSPO-PET radiotracers bear limitation such as high lipophilicity, off-target binding, poor binding to mutated TSPO isoforms.^117^ There is substantial effort to overcome such issues by synthesizing PET-TSPO ligands with moderate affinity for the target and by developing tailored approaches to data analysis.^116^ Alongside brain imaging studies, possible involvement of neuroimmune interactions in nociceptive, neuropathic, and nociplastic pain in humans is being evaluated by quantifying biomarkers of inflammation in the cerebrospinal fluid (CSF). Analysis of cytokines levels dominates the field based on the assumption that activated (micro)glia would be the major source of release and accumulation in the CSF. Levels of 21 cytokines are higher in CSF samples of people with pain compared with people without pain. However, based on few studies that attempted to correlate CSF levels with intensity of pain, such correlation cannot be conclusively drawn.^118^ Nevertheless, a promising outcome is that IL-8 (functionally overlapping with the properties of mouse CXCL1) levels are higher in nociceptive pain but not in neuropathic pain, suggesting the existence of a biomarker that can reliably distinguish between these 2 types of chronic pain.

Overall, clinical evidence from cytokine studies of the CSF and from neuroimaging studies on chronic pain conditions suggest that altered neuroimmune interactions are likely to occur in humans with validation from more controlled conditions afforded by rodent models. However, other approaches are required and, in this context the Danish Pain Research Biobank has recently collected CSF samples from pain-free volunteers (*n* = 70), people with high-impact pain (*n* = 201) and people with osteoarthritis pain (81) and they are performing biomarker analysis.^119^ The inclusion of samples from people without pain in the experimental design represents a great addition as these will provide biomarker levels against which compare levels measured in samples from people with pain. Although demographic variables such as age may exert confounding effects, we eagerly await publication of results, which will assess whether, and which, neuroimmune biomarkers are present in the CSF of people with pain.

## VI Neuropathology of Alzheimer disease in regions associated with pain processing

Considering that the AD brain pathology may impact on pain processing, we have gathered clinical evidence that AD pathology features neuronal changes and immune cell activation in brain areas involved in pain processing and discuss the implications of these findings. The AD brain features A*β*-containing extracellular plaques and tau-containing intracellular NFTs accompanied by astrogliosis and microglia cell activation.^120^ A*β* peptides that form extracellular plaques derive from APP, a single transmembrane protein that is enriched in neuron plasma membranes.^121^ APP cleavage by the *α*-secretase releases soluble APP*α* in the extracellular environment (nonamyloidogenic pathway).^121^^,^^122^ Alternatively, APP cleavage by the *β*-secretase, *β*-site APP-cleaving enzyme 1 into soluble APP*α* followed by *γ*-secretases leads to generation of A*β*peptides that are prone to aggregation (amyloidogenic pathway).^123^

Furthermore, A*β* oligomers are sufficient to induce abnormal tau protein phosphorylation leading to dystrophic neuritis and neurodegeneration in AD.^124^ Tau protein is normally present in cytoplasm of axons, presynaptic and postsynaptic compartments and associated with nuclear membranes^125^ and tau main function is thought to be microtubule stabilization.^126^ Tau protein is subject to numerous post-translational modifications that alter its physiological properties and can induce pathogenic self-aggregation and accumulation of hyperphosphorylated forms in cell bodies and dendrites.^23^^,^^127^ Neuronal loss parallels the distribution of NFTs in AD and better correlates with cognitive deficits than tau burden.^23^^,^^128^

In the human brain, the presence of AD-associated neuropathology in key pain regions has led to the assumption that this may underlie changes in pain perception (Fig. 4). In AD, histological changes can be detected in the spinal cord, the medial system region, which is involved in the emotional-affective and cognitive components of pain^129^ and lateral pain systems for sensory-discriminative aspect of pain. Concerning the spinal cord, phosphorylation of tau occurs in early stages of AD, before dementia diagnosis^108^^,^^130^ and neuropil threads and NFTs occur especially in late AD (Braak stage V–VI).^131^^,^^132^

**Fig. 4:**
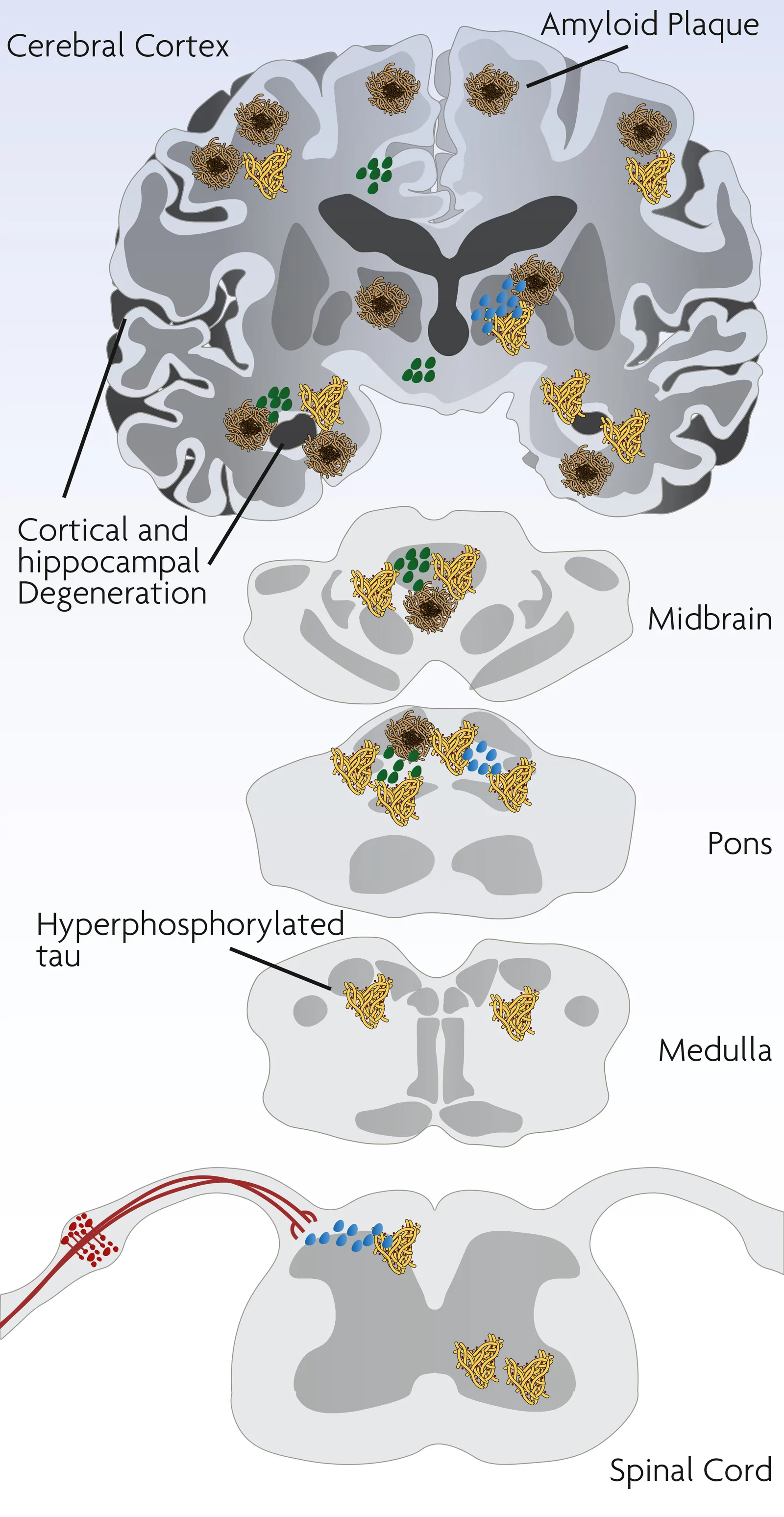
Alzheimer disease neuropathology and pain processing. Amyloid plaques and NFTs exhibit distinct and progressive spreading patterns in AD. Although AD has traditionally been considered a brain-specific disorder, emerging clinical evidence shows that neurofibrillary tau tangles can also be detected in the dorsal and ventral horns of the spinal cord, underscoring the need for more comprehensive studies on spinal cord involvement in AD. In the brainstem, regions such as the medulla, pons, and midbrain primarily accumulate hyperphosphorylated tau aggregates, with amyloid plaques typically emerging at later stages of the disease. Notably, brainstem nuclei such as the LC and dorsal raphe nucleus display early tau pathology, which may disrupt descending modulatory pathways and influence pain processing. In more advanced stages, extensive amyloid and tau pathology, along with significant neurodegeneration, are observed in the thalamus, amygdala, and insular cortex, all of which regions are integral to emotional and sensory pain processing. Interestingly, the primary somatosensory cortices are affected only in the later stages of AD, suggesting that the sensory-discriminative aspect of pain perception may remain relatively preserved until the disease is advanced.

The medial system includes the spinothalamic tract that projects directly from spinal cord to intralaminar thalamic nuclei, the spinoreticular tract that projects to parabrachial nucleus and LC, and the spinomesencephalic tract that projects to periaqueductal gray.^133^

In the AD brain there is evidence of tau pathology in the thalamus,^28^ and amyloid plaques that follow NFT formation in the intralaminar nuclei of the thalamus.^134^ Similarly, PET neuroimaging using ^11^C-PiB reveal high levels of fibrillary A*β* and reduced glucose metabolism in the thalamus of individuals with AD.^135^ Furthermore, NFTs and neuropil threads are detected in brainstem areas such as the periaqueductal gray and the parabrachial nucleus as early as Braak stage I to II, highlighting that these regions are affected very early with possible impact on the modulation of nociceptive input from the spinoreticular and spinomesencephalic tracts, respectively.^136^ The integrity of the LC is also disrupted very early in AD, with loss of LC-noradrenergic neurons in autopsy specimens of patients with AD estimated to be more than 50%.^137^, ^138^, ^139^ The lateral pain pathways include spinothalamic neurons that project from the spinal cord project to lateral thalamus and then to primary somatosensory area (S1), S2, parietal operculum, and insula.^129^^,^^133^ The primary somatosensory areas are devoid of pathology in the early stages of AD and, in fact, extensive tau pathology in the form of neuropil threads is detected only in late stages (Braak stage VI).^28^

Altogether, these clinical observations have led to the suggestion that the sensory-discriminative aspect of pain may be preserved in AD, supporting some experimental evidence that detection of noxious stimulation remains unaltered in people with AD.^59^^,^^129^ However, AD pathology in the medial pain system region has suggested that amyloid and tau aggregates may affect the emotional and cognitive components of pain.^129^ On these bases, one could hypothesizes that people with AD detect pain normally yet are unable to report pain and seek for help. There are some caveats for this simplified conclusion though. We caution that postmortem analyses should not be readily used to infer alterations in pain perception. We believe that the existence of pathology in a single region is not sufficient to infer effects on high-order functions. We hereby suggest that a successful assessment of the effects of progressive accrual of AD-related pathology in the brain should use longitudinal clinical imaging, blood plasma biomarkers combined with pain assessment including measurement of unpleasantness.

### A Inflammatory component to Alzheimer disease: Astrocytes and microglia

A feature of the AD pathology are clusters of microglia and astrocytes around amyloid plaques where these cells display a shift in phenotype from homeostatic roles to reactive states.^32^ Clinical and preclinical evidence suggest that both microglia and astrocytes play critical roles in AD pathology, and below we discuss such findings with a special weight on microglial responses some of which are also involved in nociceptive signaling.

#### 1 Astrocytes

Astrocytic responses in AD depend on disease stage: at early stage, astrocytes become reactive and protect brain tissue, at advanced AD stages, glial paralysis contributes to brain tissue atrophy, which manifests in dementia.^140^ Reactive astrocytes closely associate with amyloid plaques and create a protective barrier.^141^ Cells display morphological changes and, in the advanced stages of AD, functional asthenia (limited reactivity) in the most vulnerable regions.^140^

Astrocytes are involved in the clearance and degradation of A*β* plaques^141^ and preclinical evidence in rodents indicates that astrocytes can uptake and degrade A*β*^142^ possibly through apolipoprotein E (ApoE)-mediated internalization and subsequent degradation of A*β* peptides.^143^ Furthermore, human astrocytes that are in the close vicinity to amyloid plaques show higher expression of A*β* degrading enzymes such as neprilysin and insulin-degrading enzyme.^141^ Astrocyte-mediated protective effects in AD include inhibition of activated microglia. Preclinical evidence indicates that A*β* peptide induces the release of transforming growth factor *β*, which inhibits inducible nitric oxide synthase activity in microglia. This mechanism results in attenuation of neurotoxic effects of nitric oxide.^144^ However, continuous accumulation of A*β*-containing neuronal debris in astrocytes induces loss of homeostatic functions and preclinical observations include disturbance of glucose metabolism and impaired regulation of glutamate, which lead to impairment of neuronal viability and excitotoxicity, respectively.^144^ Additionally, A*β*-induced activation of astrocytes leads to secretion of TNF-*α* and IL-1*β*, which can cause neuronal damage.^120^ In animal models of AD, reactive astrocytes increase expression of monoamine oxidase B, which results in (1) de novo synthesis of GABA that exerts tonic inhibition of neurons and (2) production of excessive hydrogen peroxide, which causes glial activation, tauopathy, and neuronal death.^145^

Overall, astrocytes undergo functional alteration in early and late AD states, and this may affect the disease. Studies in rodents suggest significant contribution of astrocytes to AD pathology. However, human astrocytes are more complex than rodent astrocytes and display distinct calcium responses. There are also similarities and shared activities; for instance both rodent and human astrocytes promote neuronal survival and synapse formation.^146^ Current accepted knowledge in AD research suggests that astrocytes play a functional role in the development of the disease; we await better elucidation of whether they offer approaches for addressing this disease, including the provision of biomarkers.^24^

#### 2 Microglia

Similar to astrocytes, microglia accumulate in the vicinity of amyloid plaques in brains of AD individuals at autopsy and in animal models of AD.^147^ Microglia are involved in AD pathogenesis and progression^32^ and clinical evidence suggests that microglia mediate the therapeutic effects of the recently approved A*β* targeting immunotherapies.^148^

A*β* is usually removed from the brain in different ways, including export into the CSF and the blood vessels as well as by local degradation by microglial cells. Although CSF and blood drainage of A*β* each account for approximately 25% of its removal, the remaining 50% is taken up and degraded by microglia.^149^ Although microglial activation in AD is linked to disease pathophysiology, it remains unclear whether this activation is harmful or beneficial. The presence of heterogeneous microglial responses reflects stage-specific features of the disease, whereas presence of tau accumulation and amyloid deposition reveals that the same microglial pathway might have opposite results in different contexts.^150^ A great example of such disparity is represented by the CX3CL1-CX3CR1 signaling pair in the APPPS1 mouse models of AD. More specifically, disruption of CX3CL1-CX3CR1 signaling in APPPS1/*Cx3cr1*^*−/−*^ and APPPS1/*Cx3cl1*^*−/−*^ mice leads to reduction of amyloid pathology and proinflammatory cytokine levels suggesting that block of this signaling pair might be effective against amyloidosis.^151^ In contrast, deletion of *Cx3cr1* in hTau mice (Table 2^95^^,^^152^, ^153^, ^154^, ^155^, ^156^, ^157^, ^158^, ^159^, ^160^, ^161^) results in aggregation of hyperphosphorylated tau, worsened cognitive function, and increased IL-1*β* release.^162^ These preclinical findings suggest that microglia pathways implicated in AD neuropathology might distinctly regulate amyloid and tau toxicity.

**Table 2:** Transgenic mouse models of AD: AD-like pathology, sensory and motor changes, and cognitive deficits

Transgenic Mice	Type of Pathology	Behavioral Assays for Pain	Mechanism	Motor Alterations	Cognitive Defects	Studies
TASTPM Double transgenic	Amyloid deposits no tangles; microgliosis and reactive astrocytes in hippocampus, thalamus and cortex at 6 mo of age. Amyloid deposits in spinal cord at 12 mo of age	Reduced sensitivity to acute heat stimuli Reduced sensitivity to mechanical stimuli in models of chronic pain	Increased opioidergic tone Emergence of dorsal horn microglia, which is unresponsive to ATP and neuron-derived galectin-3	Decreased spontaneous activity. Intact motor coordination	From 6 mo of age	Aman et al^152^ Pugh et al^153^ Aman et al^154^ Silva et al^155^ Sideris-Lampretsas et al^95^
CRND8 transgenic mice (TgCRND8)	Amyloid deposition in the brain at 3 mo of age A*β* peptide in sensory neurons	Reduced sensitivity to thermal and mechanical stimuli	Reduced levels of pronociceptive peptides in sensory neurons	Not tested	From 3 mo of age	Shukla et al^156^
H Tau. P301S (hTau)	Tau pathology, neurofibrillary tangles at 6 mo of age and neuronal loss	Hyposensitivity to mechanical stimuli in young mice. Heat hypersensitivity at 10 m of age	Hyperphosphorylated tau aggregate in sensory neurons; reduced axonal transport of mitochondria disruption of axonal conduction	Hyperactivity; motor coordination deficits from 4 mo of age	From 3 mo of age	Mellone et al^157^ Lopez-Gonzalez et al^158^ Takeuchi et al^159^
5xFAD	Amyloid deposits at 3 mo of age and tangles by 12 mo of age	Heat and cold sensitivity unaltered at 12 mo of age	Not investigated	Normal	Between 3 and 6 mo of age	Oddo et al^160^ Baeta-Corral et al^161^

A further example is the TREM2 pathway in disease-associated microglia.^163^ TREM2 promotes microglial phagocytosis, modulates inflammatory signaling, and promotes microglial metabolic fitness and survival.^164^ TREM2 binds to soluble oligomers of A*β* and other damage-associated lipids and promotes phagocytosis.^165^ Mouse models of amyloidosis with haploinsufficiency or deletion of *Trem2,* suggest a dual role for TREM2 in amyloid accrual including decreased *Αβ* levels before plaque deposition at 2 months of age, and increased levels at 8.5 months of age after extensive plaque formation occurs.^166^ Notably, there is also reduction in the number of plaque-associated microglia, impairment in plaque compaction and increased levels of dystrophic neurites around plaques.^167^ Either TREM2 knock out mice or TREM2 R47H (R47H impairs microglial response) expression in mice that develop amyloid plaques leads to enhanced amyloid seeding as well as enhanced tau seeding and spreading near sites of amyloid deposition.^168^ In contrast, in PS19 mice (tauopathy model) TREM2 deletion significantly attenuates tau-mediated neuronal death and astrogliosis.^169^ These preclinical findings suggest that the TREM2 pathway in microglia contributes to neurodegeneration in models of tauopathy.

Microglial response to amyloid deposition and tau hyperphosphorylation includes changes in cellular transcriptome.^170^^,^^171^ In the prefrontal cortex of people with AD, microglial transcripts that are significantly downregulated around amyloid plaque include *CX3CR1*, and oppositely, transcripts such as *APOE*, *TREM2*, *AXL*, and *LGALS3* are significantly upregulated in microglia clustering *Αβ* plaques. Most of these transcripts are associated with microglial responses to increased neuronal activity and they are actively involved in nociceptive signaling in the spinal cord. In addition, microglial phagocytosis is impaired in mouse models of AD with microglia engulfing and degrading synapses,^172^ a process that affects the excitation/inhibition balance and might be involved in the hyperactivity observed in the early stages of AD.^173^ This is an important point as even though spinal microglia follow similar molecular changes in the spinal cord of a mouse model of AD,^95^ the state of dorsal horn network remains largely unexplored.

In the following section we report on a clinical study that proposes a link between microglial response to neurodegeneration and microglial response to increased neuronal activity in chronic pain states and then highlights the importance of microglial activation in pain in AD. Notably, neither preclinical nor clinical evidence yet exist for a role of reactive astrocytes in pain in AD.

## VII Microglia in the cross section of neurodegeneration and chronic pain

Relevant to this review, in people with impaired cognitive function, there is evidence of an association of chronic pain with CSF biomarkers of neurodegeneration, microglia activation, and inflammation.^174^ Specifically, using the Alzheimer’s Neuroimaging Initiative database, healthy controls were classified as individuals independent in daily living and no cognitive impairment and individuals with neurodegeneration were selected by the presence of amyloid deposition, tau pathology and neurodegeneration (magnetic resonance imaging and positron emission tomography) (ATN classification). Chronic pain individuals, those reporting persistent pain at baseline (61%), showed higher CSF levels of TNF-*α* than healthy controls independently from the ATN classification. Longitudinal analysis (baseline and after 24 months) of possible chronic pain association with CSF neurodegeneration biomarkers and memory function, revealed no interaction for chronic pain and cognitive status. However, chronic pain was associated with increased CSF levels of t-tau (total tau; marker of neurodegeneration) and sTREM 2 (marker of microglial activation) in the group of individuals with neurodegeneration (CSF t-tau ≥93 pg/ml). Thus, the increase in sTREM2 levels on one hand support the notion that microglia are activated in chronic pain states on the other hand it suggests a link between neurodegeneration and microglial activation in chronic pain states.^174^ Overall, these results suggest that microglial activation in the presence of neurodegeneration may affect microglia ability to respond to increased neuronal activity. Considering that neuronal excitation characterizes chronic pain states, microglia may provide potential therapeutic targets for chronic pain and neurodegeneration, including AD (Fig. 3).

In summary, activation of the innate immune system, and specifically activation of microglia, play a pivotal role in AD pathophysiology and these cells are implicated in the cross section of chronic pain and neurodegeneration.^174^ Nevertheless, preclinical data in AD models suggest possible divergent effects of microglia depending on disease stage and context and suggest that this conclusion should be supported by more experimentation. Future studies will need to characterize the expression profiles of microglia from both amyloid- and tau-based animal models using single-cell techniques. This data should be matched with functional experiments to identify the relevant output of a specific transcriptional profile and determine whether outcomes can be therapeutically exploited.

## VIII Pharmacotherapy of pain in Alzheimer disease

The complexity of chronic pain in individuals with dementia necessitates a comprehensive pain management approach that encompasses effective pharmacotherapy combined with nonpharmacological management. Despite such clinical need, the availability of suitable or targeted pharmacological treatments to manage chronic pain in AD remains insufficient and not fully exploited.^4^^,^^15^ Overall, patients with AD are prescribed fewer analgesics such as nonsteroidal anti-inflammatory drugs (NSAIDs) compared with cognitively intact individuals.^19^^,^^175^ In contrast, register-based cross-sectional studies from Scandinavia report that a higher proportion of individuals living with dementia use paracetamol, antipsychotics, and opioids.^176^^,^^177^ These results about opioid prescription in AD are surprising as there is evidence from 2 independent studies showing that people living with AD and needing opioids during recovering from hip fracture surgery, in reality receive 3 times less opioid analgesics than cognitively intact individuals.^178^^,^^179^

The principal treatment for mild to moderate pain in AD is paracetamol with additional use of NSAIDs, the latter appear to be associated with increased risk of gastrointestinal and cardiovascular adverse effects, and some NSAIDs may also cause hepatic and renal damage.^180^ However, there is a substantial paucity of clinical studies for the treatment of pain in AD. In particular, some double-blinded randomized controlled trials (RCTs) have evaluated paracetamol versus placebo in people with dementia, whereas there are no trials for the analgesic effect of antidepressants and antiepileptics.^4^^,^^42^ Paracetamol appears a safer alternative medication to atypical antipsychotics.^180^, ^181^, ^182^ In a study of stepped pain treatment for agitation, paracetamol improved agitation and pain in residents of nursing homes with moderate to severe dementia and was well tolerated.^183^ Concerning RCTs on the use of opioids, one study reported that 23 of the 44 patients withdrew transdermal buprenorphine because of adverse events.^184^ The most frequent adverse effects were psychiatric and neurologic and included personality changes, confusion, and sedation. Thus, despite their efficacy opioid analgesics should be prescribed with caution owing to their psychiatric adverse effects and their potential to aggravate dementia. A recent meta-analysis has reported that out of more than a million individuals with chronic noncancer pain, opioid users had 15% higher risk of developing dementia than the control group.^185^ The challenges faced with using opioids and NSAIDs, highlight the need for new generation therapeutics for pain not responding to paracetamol.

Altogether, the limitation of most of these RCTs is that they usually include few participants and short follow-ups; in addition, these studies apply inclusion and exclusion criteria, which limit generalizability to the patient population. Further RCTs are required to create evidence-based treatment guidelines and to provide mechanistic insights into the putative perturbation of nociceptive signaling in AD. Such clinical trials will need to consider cognitive side effects, polypharmacy concerns that may lead to inappropriate prescribing, regulatory challenges that foster the development of safe and effective medicine for people with AD,^186^ and optimal dosing in the elderly with dementia.^187^

## IX Nociception in animal models of Alzheimer disease

Aiming to address the challenge presented by AD, animal models constitute a vital tool to study the pathophysiological mechanisms underlying the onset and progression of the disease. However, such models of either amyloid or tauopathy do not reproduce the complexity of the AD pathology and there is a continuous effort to improve their translational value into the clinic.^188^

The advent of new technologies for the generation of transgenic mice in combination with the long list of failed human AD trials has propelled the field to create a set of different rodent models of AD that can recapitulate several of the human AD pathological hallmarks, including amyloid plaque formation, tau hyperphosphorylation and memory deficits (Table 2).^189^ In addition to cognitive performance, mouse models of AD are being used to identify possible changes in nociceptive processing. Below, we provide an overview of preclinical studies exploring the interaction between AD neuropathology and mechanisms underlying acute and chronic pain. We examine each mouse model individually, first explaining how models replicate key features of AD, and then discussing how their use has contributed to our understanding of changes in sensory mechanisms in the presence of AD-like pathology. In these models a point of caution is due to the interpretation of pain-like behavior in older transgenic mice, which may be influenced by age and specific phenotypes. However, the inclusion of age-matched control groups can mitigate for possible influence of age on nociceptive thresholds. Nevertheless, features such as hyperactivity can result in unreliable threshold values and we discuss below when this becomes a confounding factor.

Most preclinical studies investigating nociception in AD use the TASTPM mouse model, which is a double transgenic mouse carrying 2 mutations associated with early-onset disease (Table 2).^190^ TASTPM mice show brain deposition of amyloid plaques in cortex, thalamus and hippocampus and intraneuronal APP/A*β* accumulation in the dorsal and ventral gray matter of the spinal cord at 6 months of age. By 12 months of age, amyloid plaques can be detected in the spinal cord and APP is found in intraneuronal structures in the dorsal and ventral horn of the lumbar spinal cord.^152^ In concomitance to age-dependent pathological features, from 6 months of age TASTPM mice of both sexes display cognitive deficits in the novel object recognition test and decreased spontaneous motor activity, but no alteration in performance on the accelerating rota-rod apparatus.^153^ This intact ability to coordinate motor behavior on the rota-rod excludes possible interference with hind paw withdrawal thresholds in nociceptive behavior tests. The presence of cognitive deficits is unlikely to influence hind paw withdrawal, which is not a learned behavior.^191^ Six months old TASTPM mice show some degree of tolerance to noxious heat maintained by an endogenous opioid-mediated tone.^152^ Such an inhibitory tone also attenuates the extent and duration of mechanical allodynia in models of neuropathic and osteoarthritis chronic pain in which TASPM mice exhibit elevated plasma levels of *β*-endorphin and greater levels of morphine-induced analgesia. Indeed, in comparison to wild-type, TASTPM spinal cord show higher neuronal expression of enkephalins that exert neuronal inhibition and modulate nociceptive signaling at dorsal horn level.^154^^,^^155^ These observations correlate with evidence that people with AD show higher CSF levels of opioid peptides.^192^ In addition, in models of chronic inflammatory pain, compared with wild-type, new subsets of microglia emerge in the TASTPM spinal cord and mice respond less to nociceptive signaling. Specifically, TASTPM dorsal horn microglia significantly downregulate expression of P2Y12R and TLR4 receptors, both of which are essential for microglia to respond to noxious stimulation-evoked release of ATP and sensory neuron-derived Gal-3, respectively.^95^ Overall, our observations in the TASTPM mouse model of AD point to the existence of alterations in nociceptive processing in chronic pain states, and suggest that there is an attenuated pain-like behavior, which correlates with disruption in the opioidegic system and alteration of microglia responsiveness in the spinal cord.

Like TASTPM, TgCRND8 mice (Table 2) show reduced sensitivity to thermal and mechanical stimulation. These mice develop early plaque formation and cognitive impairment at 3 months of age.^193^ Hyposensitivity to noxious thermal and tactile stimulation starts at 3 months and persists at 12 and 24 months of age. In addition, 3-month-old mice are less susceptible to the development of inflammatory nociception in the intraplantar complete Freund’s adjuvant model.^156^ However, differently from TASTPM, TgCRND8 mice pathology affects sensory neurons in which the expression of nociceptive peptides such as calcitonin-gene-related peptide and substance P appear downregulated and explains the attenuated response of sensory neurons to noxious stimuli. Since whether AD is associated with sensory neuron pathology remains to be established, these observations may bear limited translational value. Another mouse models of A*β* amyloidosis generated by knock-in of a humanized A*β* sequence with 2 or 3 human APP mutations (*App*^*NL-F,*^ *App*^*NL-G-F*^) display plaque development as early as at 2 months of age and behavioral changes associated with anxiety-related behavior before the onset of cognitive deficits.^194^ Sensory tests have not yet been performed in these mice, though 15 to 17-month-old *App*^*NL-G-F*^ mice display altered responses to aversive stimulation such as hyperactivity to a mild foot shock that can be interpreted as a reaction to painful stimulation.

The involvement of tau hyperphosphorylation in nociceptive signaling is confined to the P301S model. This transgenic mouse does not develop amyloid plaques but rather constitutes the most used model to study the effects of NFTs, which are present from 6 months of age. Importantly, in contrast to models of amyloidosis where neuronal loss is not observed, the P301S mice show marked neuronal loss in the cortex, amygdala, and hippocampus, thus recapitulating neurodegeneration observed in patients with AD.^195^ Regarding nociceptive signaling, data on whether tau pathology is associated with sensory alterations is more ambiguous and includes evidence for hyposensitivity in young P301S mice and hypersensitivity in older mice. In young and adult mice there is an age-dependent reduction in mechanical sensitivity but no changes in thermal nociception.^157^ Instead, 6- and 10-month-old P301S mice show heat hypersensitivity, which is associated with elevated levels of cytokines in somatosensory cortex, hippocampus, and lumbar spinal cord, suggesting that dysregulation of the immune response might cause alterations in responses to heat stimulation.^158^^,^^159^ These findings could be impacted by mice hyperactivity, which characterizes this human Tau model. Furthermore, in tau transgenic mice DRG neurons express human P301S tau, which can be an underlying mechanism for sensory changes. In PNS neurons, tau pathology develops in similar fashion to CNS neurons and axonal transport of mitochondria appears to be affected by the formations of hyperphosphorylated tau aggregates in DRG neurons.^157^ Indeed, in tau-null mice, congenital loss of tau results in decreased density of unmyelinated C fibers and hypomyelination of A*δ*-fibers in sciatic nerves.^196^ Intriguingly, changes in myelination and primary afferent nerve density affect neuronal functional outputs with changes in compound action potentials of myelinated versus unmyelinated fibers indicating a disruption in conduction properties of sciatic nerve in tau-null mice.

Finally, to address the limitations of using amyloid or tau-only mice, there is the triple-transgenic mouse (3xTg) model (Table 2). Mice display plaques as early as 3 months of age and tangles by 12 months of age.^160^ In 2 independent studies, heat and cold sensitivity appears unaltered in 12-month-old 3xTg mice.^160^^,^^161^ Nonetheless, here we caution that the primary focus of these studies was not an in-depth characterization of nociception and there is no molecular and cellular characterization of AD-like pathology in regions involved in nociception. Thus, more studies are required on the combined effect of amyloidosis and tau hyperphosphorylation in peripheral and central pain processing using this mouse model tool.

In summary, TASTPM and TgCRND8 mice share hyposensitivity to noxious stimuli despite displaying pathology in CNS only (increased opioidergic tone and altered microglial responsiveness) and CNS and PNS pathology (peptide content changes in peripheral sensory neurons), respectively. P301S mice show hyposensitivity at young age followed by hypersensitivity in older mice and tau pathology in both CNS and PNS. The 3xTg mice display both plaques and tangles and no sensory changes. However, in both P301S0hTau and 3xTg mouse models, alterations in motor behavior might influence quantification of hind paw withdrawal thresholds and assessment of sensory changes.

Collectively, this evidence indicates transgenic mouse models are an invaluable tool to study of the mechanisms governing the alteration of nociception present in AD. We reason that more mechanistic studies are necessary, ideally with a focus on defining spinal cord circuits in AD models.

## X Experimental therapeutics: The case of galectin-3

Microglia activation is a driving force in the pathogenesis and progression of both chronic pain and AD. The work we have reviewed here indicates that microglia offer targets that might be beneficial in both conditions. Such targets would need to demonstrate a role in determining the pathologies and should be validated in the clinical settings. Here, we propose that Gal-3 meets these criteria and holds the potential of a therapeutic target for both chronic pain and AD for the following reasons. Firstly, Gal-3 levels increase in AD brain parenchyma, CSF and plasma: Gal-3 levels closely follow levels of p-Tau181 and neurogranin, surrogate markers of neuronal death and synapse degeneration.^165^^,^^197^, ^198^, ^199^ Secondly, in the CNS, Gal-3 is mostly expressed by microglia and *Lgals3*, the gene encoding for Gal-3, is a highly upregulated transcript in mouse microglia in response to amyloid^200^, ^201^, ^202^ and detected in microglia adjacent to A*β* plaques and NFTs in AD brains.^203^^,^^204^ Thirdly, preclinical evidence suggests that Gal-3 promotes A*β* and phosphorylated Tau oligomerization through binding to TREM2 and TLR4,^203^^,^^205^^,^^206^ and in the spinal cord, Gal-3 activation of TLR4 mediates inflammatory and neuropathic pain mechanisms.^95^^,^^207^^,^^208^ In detail, deletion of *Lglas3* in 5xFAD mouse model of AD reduces plaque pathology, ameliorates memory deficits, and attenuates microglial responses associated with TLR4 and TREM2 signaling.^203^ In the spinal cord, endogenous Gal-3 levels increase following peripheral tissue/nerve damage and intrathecal administration of recombinant Gal-3 produces mechanical allodynia.^95^^,^^208^^,^^209^ Moreover, blockade of spinal Gal-3 attenuates both arthritis and neuropathic pain-like behavior, suggesting that Gal-3 exerts a pronociceptive action, whereas approaches to inhibit Gal-3 alleviate chronic pain.^95^^,^^208^^,^^210^ In spinal cord dorsal horn, Gal-3 is released by primary afferent terminals, activates TLR4 on microglia promoting release of proinflammatory cytokines that facilitate nociceptive signaling.^95^ Such pronociceptive pathway is perturbed in the TASTPM mouse model of AD, where a subset of spinal microglia lacks TLR4 and are unable to respond to Gal-3. This anomaly results in lower susceptibility to develop chronic pain by TASTPM mice.

Thus, these observations point to Gal-3 as a candidate target across AD and chronic pain. Furthermore, Gal-3 inhibition would be desirable in heart failure and tissue fibrosis as well as in certain form of cancers,^211^^,^^212^ though Gal-3 inhibition might be detrimental after brain ischemia where Gal-3 plays a neuroprotective role.^213^ Despite significant effort, only few Gal-3 inhibitors have entered clinical trials; these studies, when reported, have indicated poor selectivity and potency yet no safety issues have been raised.^214^

It is important to note that additional immune targets, such as CSF1R and IL-1*β*, have demonstrated neuroprotective and analgesic effects in preclinical models.^215^, ^216^, ^217^

However, immune targets involvement in nociception within the context of AD remains unclear. Specifically, mouse microglia activated by CSF/CSF1R can degrade A*β*^218^ and genetic screening in patients with AD has revealed presence of CSFR1 variants in late-onset AD.^219^ In the APP/PS1 mouse model of AD, inhibition of CSF1R decreases microglial proliferation in proximity to A*β* plaques, and reduces degeneration of synapses, without modifying the levels of A*β*.^220^ Inhibition of CSF1R reduces the expression of IL-1*β* and TNF*α* and blockade of microglial proliferation attenuates neuronal degeneration and ameliorates tau pathology.^221^ Thus, reduction of microglial proliferation and microglial depletion provide an opportunity to control AD neuropathology. So far, clinical investigations on CSF1/CS1R pair include a phase 1 randomized placebo controlled, single-bind study in people with mild cognitive impairments to characterize biomarker effects of a colony-stimulating factor 1 receptor antagonist, JNJ-40346527 (ClinicalTrials.gov ID NCT04121208). If biomarkers are identified in the study, the aim is to test whether JNJ-40346527 can slow or prevent the progression of AD.

Other possible immune targets include NLP3 and IL-1*β*. Microglial response to A*β* includes activation of the NOD-like receptor (NLR) family, pyrin domain containing 3 (NLP3) inflammasome. Preclinical evidence in mouse models of AD, shows that A*β* binding to TLR activates NLP3, which leads to generation of IL-1*β* that can induce neuronal degeneration. Instead, APP/PS1xNLP3 knockout mice, which are genetically deficient for NLR family pyrin domain containing 3 (NLRP3), send processes into A*β* deposits and have a twofold increase in phagocytic clearance capacity. This evidence has led to the proposal that inhibition of inflammasome activation and IL-1*β* may offer therapeutic avenues.^222^

Overall, there is an encouraging effort at preclinical level at identifying innate immunity targets for chronic pain and for AD separately. We propose Gal-3 inhibition as a dual approach for AD and chronic pain. On a final note, a recent phase 1b/2 proof-of-concept trial of TB006, a well tolerated investigational monoclonal antibody against Gal-3 for the treatment of mild to severe AD, showed modest improvement of dementia (CDR-SB numeric scale used to quantify severity of symptoms of dementia).^223^ However, TB006 improved mini mental state examination and reduced plasma *Αβ*42 levels (https://static1.squarespace.com/static/6118e5173909f118e816918e/t/637871ba8ec03853236e7317/1668842422452/TrueBinding-Topline-Release.pdf). This exciting data points toward some clinical evidence for Gal-3 relevance in the pathophysiology of AD. It remains to be established whether treatment with TB006 exerts positive effects on chronic pain including pain in people with AD.

## XI Conclusion

Despite chronic pain conditions being common in people living with AD, analgesics are under prescribed in this patient population because of a deficit in reporting symptoms. AD neuropathology alters both pain assessment/identification in humans and nociceptive thresholds in transgenic mouse models of AD. Thus, clinical observations suggest that pain tolerance and affective modulation of pain may be altered in AD. Preclinical data reveal subtle alterations in sensory thresholds to acute heat and reduced susceptibility to develop chronic pain, which are associated with heighten inhibitory opioid tone and reduced levels of pronociceptive peptides in sensory neurons. Notably, both clinical and preclinical evidence indicates that chronic pain and AD share mechanisms that involve microglia activation and Gal-3 that link both conditions and are potential therapeutic targets. It is tempting to suggest that pain is a *silent epidemic* among people living with dementia and key research priorities include prospective human imaging cohorts, longitudinal pain assessments, and combined pain and AD clinical trials. In addition, a better understanding of the complex interplay between chronic pain and AD pathophysiology offers opportunities to identify novel approaches to combat neurodegeneration and jointly diagnosed chronic pain.

## Conflict of interest

The authors declare no conflicts of interest.

## References

[1] Basbaum A.I., Bautista D.M., Scherrer G., Julius D. (2009). Cellular and molecular mechanisms of pain. Cell.

[2] Cox J.J., Reimann F., Nicholas A.K. (2006). An SCN9A channelopathy causes congenital inability to experience pain. Nature.

[3] Drissi I., Woods W.A., Woods C.G. (2020). Understanding the genetic basis of congenital insensitivity to pain. Br Med Bull.

[4] Achterberg W., Lautenbacher S., Husebo B., Erdal A., Herr K. (2020). Pain in dementia. Pain Rep.

[5] Sandkuhler J. (2009). Models and mechanisms of hyperalgesia and allodynia. Physiol Rev.

[6] Gilron I., Baron R., Jensen T. (2015). Neuropathic pain: principles of diagnosis and treatment. Mayo Clin Proc.

[7] Cohen S.P., Vase L., Hooten W.M. (2021). Chronic pain: an update on burden, best practices, and new advances. Lancet.

[8] Finnerup N.B., Kuner R., Jensen T.S. (2021). Neuropathic pain: from mechanisms to treatment. Physiol Rev.

[9] Walsh D.A., McWilliams D.F. (2014). Mechanisms, impact and management of pain in rheumatoid arthritis. Nat Rev Rheumatol.

[10] Kosek E., Clauw D., Nijs J. (2021). Chronic nociplastic pain affecting the musculoskeletal system: clinical criteria and grading system. Pain.

[11] Walsh D.A. (2021). Nociplastic pain: helping to explain disconnect between pain and pathology. Pain.

[12] Fitzcharles M.-A., Cohen S.P., Clauw D.J., Littlejohn G., Usui C., Häuser W. (2021). Nociplastic pain: towards an understanding of prevalent pain conditions. Lancet.

[13] Fayaz A., Croft P., Langford R.M., Donaldson L.J., Jones G.T. (2016). Prevalence of chronic pain in the UK: a systematic review and meta-analysis of population studies. BMJ Open.

[14] Monroe T.B., Gibson S.J., Bruehl S.P. (2016). Contact heat sensitivity and reports of unpleasantness in communicative people with mild to moderate cognitive impairment in Alzheimer's disease: a cross-sectional study. BMC Med.

[15] Corbett A., Husebo B., Malcangio M. (2012). Assessment and treatment of pain in people with dementia. Nat Rev Neurol.

[16] Barry H.E., Parsons C., Passmore A.P., Hughes C.M. (2016). Exploring the prevalence of and factors associated with pain: a cross-sectional study of community-dwelling people with dementia. Health Soc Care Community.

[17] Browne J., Edwards D.A., Rhodes K.M., Brimicombe D.J., Payne R.A. (2017). Association of comorbidity and health service usage among patients with dementia in the UK: a population-based study. BMJ Open.

[18] Cohen-Mansfield J. (2008). The relationship between different pain assessments in dementia. Alzheimer Dis Assoc Disord.

[19] Bauer U., Pitzer S., Schreier M.M., Osterbrink J., Alzner R., Iglseder B. (2016). Pain treatment for nursing home residents differs according to cognitive state – a cross-sectional study. BMC Geriatr.

[20] Domenichiello A.F., Ramsden C.E. (2019). The silent epidemic of chronic pain in older adults. Prog Neuro-Psychopharmacology Biol Psychiatry.

[21] Whitlock E.L., Diaz-Ramirez L.G., Glymour M.M., Boscardin W.J., Covinsky K.E., Smith A.K. (2017). Association between persistent pain and memory decline and dementia in a longitudinal cohort of elders. JAMA Intern Med.

[22] Scheltens P., De Strooper B., Kivipelto M. (2021). Alzheimer's disease. Lancet.

[23] Serrano-Pozo A., Frosch M.P., Masliah E., Hyman B.T. (2011). Neuropathological alterations in Alzheimer disease. Cold Spring Harb Perspect Med.

[24] Arranz A.M., De Strooper B. (2019). The role of astroglia in Alzheimer’s disease: pathophysiology and clinical implications. Lancet Neurol.

[25] Madore C., Yin Z., Leibowitz J., Butovsky O. (2020). Microglia, lifestyle stress, and neurodegeneration. Immunity.

[26] Leng F., Edison P. (2020). Neuroinflammation and microglial activation in Alzheimer disease: where do we go from here?. Nat Rev Neurol.

[27] Nortley R., Korte N., Izquierdo P. (2019). Amyloid β oligomers constrict human capillaries in Alzheimer's disease via signaling to pericytes. Science.

[28] Braak H., Braak E. (1991). Neuropathological stageing of Alzheimer-related changes. Acta Neuropathol.

[29] Braak H., Braak E. (1995). Staging of Alzheimer’s disease-related neurofibrillary changes. Neurobiol Aging.

[30] Thal D.R., Rüb U., Orantes M., Braak H. (2002). Phases of Aβ-deposition in the human brain and its relevance for the development of AD. Neurology.

[31] Efthymiou A.G., Goate A.M. (2017). Late onset Alzheimer’s disease genetics implicates microglial pathways in disease risk. Mol Neurodegener.

[32] Heneka M.T., van der Flier W.M., Jessen F. (2025). Neuroinflammation in Alzheimer disease. Nat Rev Immunol.

[33] Ferretti M.T., Iulita M.F., Cavedo E. (2018). Sex differences in Alzheimer disease — the gateway to precision medicine. Nat Rev Neurol.

[34] Bellenguez C., Küçükali F., Jansen I. (2022). New insights on the genetic etiology of Alzheimer’s and related dementia. Nat Genet.

[35] Tansley S., Uttam S., Ureña Guzmán A. (2022). Single-cell RNA sequencing reveals time- and sex-specific responses of mouse spinal cord microglia to peripheral nerve injury and links ApoE to chronic pain. Nat Commun.

[36] Malcangio M., Sideris-Lampretsas G. (2025). How microglia contribute to the induction and maintenance of neuropathic pain. Nat Rev Neurosci.

[37] Sideris-Lampretsas G., Malcangio M. (2021). Microglial heterogeneity in chronic pain. Brain Behav Immun.

[38] Ji R.-R., Donnelly C.R., Nedergaard M. (2019). Astrocytes in chronic pain and itch. Nat Rev Neurosci.

[39] Kim J., Yoo I.D., Lim J., Moon J.-S. (2024). Pathological phenotypes of astrocytes in Alzheimer's disease. Exp Mol Med.

[40] Achterberg W.P., Pieper M.J.C., van Dalen-Kok A.H. (2013). Pain management in patients with dementia. Clin Interv Aging.

[41] Lawn T., Aman Y., Rukavina K. (2020). Pain in the neurodegenerating brain: insights into pharmacotherapy for Alzheimer disease and Parkinson disease. Pain.

[42] Husebo B.S., Achterberg W., Flo E. (2016). Identifying and managing pain in people with Alzheimer’s disease and other types of dementia: a systematic review. CNS Drugs.

[43] Ferrell B.A., Ferrell B.R., Rivera L. (1995). Pain in cognitively impaired nursing home patients. J Pain Symptom Manage.

[44] Malara A., De Biase G.A., Bettarini F. (2016). Pain assessment in elderly with behavioral and psychological symptoms of dementia. J Alzheimers Dis.

[45] van Kooten J., Smalbrugge M., van der Wouden J.C., Stek M.L., Hertogh C.M.P.M. (2017). Prevalence of pain in nursing home residents: the role of dementia stage and dementia subtypes. J Am Med Dir Assoc.

[46] Sampson E.L., White N., Lord K. (2015). Pain, agitation, and behavioural problems in people with dementia admitted to general hospital wards: a longitudinal cohort study. Pain.

[47] Shega J.W., Hougham G.W., Stocking C.B., Cox-Hayley D., Sachs G.A. (2004). Pain in community-dwelling persons with dementia: frequency, intensity, and congruence between patient and caregiver report. J Pain Symptom Manage.

[48] Hendriks S.A., Smalbrugge M., Galindo-Garre F., Hertogh C.M.P.M., van der Steen J.T. (2015). From admission to death: prevalence and course of pain, agitation, and shortness of breath, and treatment of these symptoms in nursing home residents with dementia. J Am Med Dir Assoc.

[49] Rajkumar A.P., Ballard C., Fossey J. (2017). Epidemiology of pain in people with dementia living in care homes: longitudinal course, prevalence, and treatment implications. J Am Med Dir Assoc.

[50] Husebo B.S., Heintz H.L., Berge L.I., Owoyemi P., Rahman A.T., Vahia I.V. (2020). Sensing technology to facilitate behavioral and psychological symptoms and to monitor treatment response in people with dementia: a systematic review. Front Pharmacol.

[51] Warden V., Hurley A.C., Volicer L. (2003). Development and psychometric evaluation of the pain assessment in advanced dementia (PAINAD) scale. J Am Med Dir Assoc.

[52] Zwakhalen S.M.G., van der Steen J.T., Najim M.D. (2012). Which score most likely represents pain on the observational PAINAD pain scale for patients with dementia?. J Am Med Dir Assoc.

[53] Husebo B.S. (2017). Mobilization-observation-behaviour-intensity-dementia-2 pain scale (MOBID-2). J Physiother.

[54] Abbey J., Piller N., De Bellis A. (2004). The Abbey pain scale: a 1-minute numerical indicator for people with end-stage dementia. Int J Palliat Nurs.

[55] Ezzati A., Wang C., Katz M.J. (2019). The temporal relationship between pain intensity and pain interference and incident dementia. Curr Alzheimer Res.

[56] Ikram M., Innes K., Sambamoorthi U. (2019). Association of osteoarthritis and pain with Alzheimer’s diseases and related dementias among older adults in the United States. Osteoarthr Cartil.

[57] Hu Y.-H., Seo D.-C., Huber L., Shih P.C., Lin H.-C. (2024). Chronic non-cancer pain and associated risks of incident mild cognitive impairment and Alzheimer’s disease and related dementias in middle-aged and older adults. J Appl Gerontol.

[58] Bornier N., Mulliez A., Chenaf C. (2023). Chronic pain is a risk factor for incident Alzheimer’s disease: a nationwide propensity-matched cohort using administrative data. Front Aging Neurosci.

[59] Benedetti F., Vighetti S., Ricco C. (1999). Pain threshold and tolerance in Alzheimer’s disease. Pain.

[60] Gibson S.J., Voukelatos X., Ames D., Flicker L., Helme R.D. (2001). An examination of pain perception and cerebral event-related potentials following carbon dioxide laser stimulation in patients with Alzheimer’s disease and age-matched control volunteers. Pain Res Manag.

[61] Cole L.J., Farrell M.J., Duff E.P., Barber J.B., Egan G.F., Gibson S.J. (2006). Pain sensitivity and fMRI pain-related brain activity in Alzheimer’s disease. Brain.

[62] Jensen-Dahm C., Werner M.U., Dahl J.B. (2014). Quantitative sensory testing and pain tolerance in patients with mild to moderate Alzheimer disease compared to healthy control subjects. Pain.

[63] Kunz M., Scharmann S., Hemmeter U., Schepelmann K., Lautenbacher S. (2007). The facial expression of pain in patients with dementia. Pain.

[64] Kunz M., Mylius V., Scharmann S., Schepelman K., Lautenbacher S. (2009). Influence of dementia on multiple components of pain. Eur J Pain.

[65] Beach P.A., Huck J.T., Miranda M.M., Foley K.T., Bozoki A.C. (2016). Effects of Alzheimer disease on the facial expression of pain. Clin J Pain.

[66] Cole L.J., Gavrilescu M., Johnston L.A., Gibson S.J., Farrell M.J., Egan G.F. (2011). The impact of Alzheimer’s disease on the functional connectivity between brain regions underlying pain perception. Eur J Pain.

[67] Jensen-Dahm C., Madsen C.S., Waldemar G. (2016). Contact heat evoked potentials (CHEPs) in patients with mild-moderate Alzheimer’s disease and matched control—A pilot study. Pain Med.

[68] Benedetti F., Arduino C., Vighetti S., Asteggiano G., Tarenzi L., Rainero I. (2004). Pain reactivity in Alzheimer patients with different degrees of cognitive impairment and brain electrical activity deterioration. Pain.

[69] Benedetti F., Arduino C., Costa S. (2006). Loss of expectation-related mechanisms in Alzheimer’s disease makes analgesic therapies less effective. Pain.

[70] Dubin A.E., Patapoutian A. (2010). Nociceptors: the sensors of the pain pathway. J Clin Invest.

[71] Peirs C., Williams S.G., Zhao X. (2021). Mechanical allodynia circuitry in the dorsal horn is defined by the nature of the injury. Neuron.

[72] Todd A.J. (2010). Neuronal circuitry for pain processing in the dorsal horn. Nat Rev Neurosci.

[73] Hunt S.P., Mantyh P.W. (2001). The molecular dynamics of pain control. Nat Rev Neurosci.

[74] Bannister K., Dickenson A.H. (2017). The plasticity of descending controls in pain: translational probing. J Physiol.

[75] Puopolo M. (2019). The hypothalamic-spinal dopaminergic system: a target for pain modulation. Neural Regen Res.

[76] De Preter C.C., Heinricher M.M. (2024). The 'in's and out's' of descending pain modulation from the rostral ventromedial medulla. Trends Neurosci.

[77] Gold M.S., Gebhart G.F. (2010). Nociceptor sensitization in pain pathogenesis. Nat Med.

[78] Schaible H.-G., Ebersberger A., Natura G. (2011). Update on peripheral mechanisms of pain: beyond prostaglandins and cytokines. Arthritis Res Ther.

[79] Woolf C.J., Ma Q. (2007). Nociceptors—Noxious stimulus detectors. Neuron.

[80] St. Pierre M., Reeh P.W., Zimmermann K. (2009). Differential effects of TRPV channel block on polymodal activation of rat cutaneous nociceptors in vitro. Exp Brain Res.

[81] Mizumura K., Sugiura T., Katanosaka K., Banik R.K., Kozaki Y. (2009). Excitation and sensitization of nociceptors by bradykinin: what do we know?. Exp Brain Res.

[82] Schaible H., von Banchet G.S., Boettger M.K. (2010). The role of proinflammatory cytokines in the generation and maintenance of joint pain. Ann N Y Acad Sci.

[83] Schweizerhof M., Stösser S., Kurejova M. (2009). Hematopoietic colony–stimulating factors mediate tumor-nerve interactions and bone cancer pain. Nat Med.

[84] Üçeyler N., Schäfers M., Sommer C. (2009). Mode of action of cytokines on nociceptive neurons. Exp Brain Res.

[85] Ji R.-R., Kohno T., Moore K.A., Woolf C.J. (2003). Central sensitization and LTP: do pain and memory share similar mechanisms?. Trends Neurosci.

[86] Woolf C., King A. (1990). Dynamic alterations in the cutaneous mechanoreceptive fields of dorsal horn neurons in the rat spinal cord. J Neurosci.

[87] Bannister K., Dickenson A.H. (2016). What do monoamines do in pain modulation?. Curr Opin Support Palliat Care.

[88] Ji R.-R., Nackley A., Huh Y., Terrando N., Maixner W. (2018). Neuroinflammation and central sensitization in chronic and widespread pain. Anesthesiology.

[89] Cserép C., Pósfai B., Dénes Á. (2020). Shaping neuronal fate: functional heterogeneity of direct microglia-neuron interactions. Neuron.

[90] Hansen R.R., Malcangio M. (2013). Astrocytes--multitaskers in chronic pain. Eur J Pharmacol.

[91] Chen G., Zhang Y.-Q., Qadri Y.J., Serhan C.N., Ji R.-R. (2018). Microglia in pain: detrimental and protective roles in pathogenesis and resolution of pain. Neuron.

[92] Inoue K., Tsuda M. (2018). Microglia in neuropathic pain: cellular and molecular mechanisms and therapeutic potential. Nat Rev Neurosci.

[93] Malcangio M. (2019). Role of the immune system in neuropathic pain. Scand J Pain.

[94] McMahon S.B., Malcangio M. (2009). Current challenges in glia-pain biology. Neuron.

[95] Sideris-Lampretsas G., Oggero S., Zeboudj L. (2023). Galectin-3 activates spinal microglia to induce inflammatory nociception in wild type but not in mice modelling Alzheimer’s disease. Nat Commun.

[96] Guan Z., Kuhn J.A., Wang X. (2016). Injured sensory neuron–derived CSF1 induces microglial proliferation and DAP12-dependent pain. Nat Neurosci.

[97] Coull J.A.M., Beggs S., Boudreau D. (2005). BDNF from microglia causes the shift in neuronal anion gradient underlying neuropathic pain. Nature.

[98] Staniland A.A., Clark A.K., Wodarski R. (2010). Reduced inflammatory and neuropathic pain and decreased spinal microglial response in fractalkine receptor (CX3CR1) knockout mice. J Neurochem.

[99] Clark A.K., Yip P.K., Grist J. (2007). Inhibition of spinal microglial cathepsin S for the reversal of neuropathic pain. Proc Natl Acad Sci USA.

[100] Clark A.K., Yip P.K., Malcangio M. (2009). The liberation of fractalkine in the dorsal horn requires microglial cathepsin S. J Neurosci.

[101] Agalave N.M., Larsson M., Abdelmoaty S. (2014). Spinal HMGB1 induces TLR4-mediated long-lasting hypersensitivity and glial activation and regulates pain-like behavior in experimental arthritis. Pain.

[102] Christianson C.A., Dumlao D.S., Stokes J.A. (2011). Spinal TLR4 mediates the transition to a persistent mechanical hypersensitivity after the resolution of inflammation in serum-transferred arthritis. Pain.

[103] Bruno K., Woller S.A., Miller Y.I. (2018). Targeting toll-like receptor-4 (TLR4)-an emerging therapeutic target for persistent pain states. Pain.

[104] Austin P.J., Fiore N.T. (2019). Supraspinal neuroimmune crosstalk in chronic pain states. Curr Opin Physiol.

[105] Taylor A.M.W., Mehrabani S., Liu S., Taylor A.J., Cahill C.M. (2017). Topography of microglial activation in sensory- and affect-related brain regions in chronic pain. J Neurosci Res.

[106] Fiore N.T., Austin P.J. (2019). Peripheral nerve injury triggers neuroinflammation in the medial prefrontal cortex and ventral hippocampus in a subgroup of rats with coincident affective behavioural changes. Neuroscience.

[107] Barcelon E.E., Cho W.H., Jun S.B., Lee S.J. (2019). Brain microglial activation in chronic pain-associated affective disorder. Front Neurosci.

[108] Gui W.-S., Wei X., Mai C.-L. (2016). Interleukin-1β overproduction is a common cause for neuropathic pain, memory deficit, and depression following peripheral nerve injury in rodents. Mol Pain.

[109] Xu N., Tang X.-H., Pan W. (2017). Spared nerve injury increases the expression of microglia M1 markers in the prefrontal cortex of rats and provokes depression-like behaviors. Front Neurosci.

[110] Loggia M.L., Chonde D.B., Akeju O. (2015). Evidence for brain glial activation in chronic pain patients. Brain.

[111] Gao Y., Zhang L., Ji R. (2010). Spinal injection of TNF-α-activated astrocytes produces persistent pain symptom mechanical allodynia by releasing monocyte chemoattractant protein-1. Glia.

[112] Wei F., Guo W., Zou S., Ren K., Dubner R. (2008). Supraspinal glial–neuronal interactions contribute to descending pain facilitation. J Neurosci.

[113] Landry R.P., Jacobs V.L., Romero-Sandoval E.A., DeLeo J.A. (2012). Propentofylline, a CNS glial modulator does not decrease pain in post-herpetic neuralgia patients: in vitro evidence for differential responses in human and rodent microglia and macrophages. Exp Neurol.

[114] Loggia M.L. (2024). "Neuroinflammation": does it have a role in chronic pain? Evidence from human imaging. Pain.

[115] Cheng J.C., Anzolin A., Berry M. (2022). Dynamic functional connectivity underlying temporal summation of pain in fibromyalgia. Arthritis Rheumatol.

[116] Turkheimer F.E., Rizzo G., Bloomfield P.S. (2015). The methodology of TSPO imaging with positron emission tomography. Biochem Soc Trans.

[117] Cumbers G.A., Harvey-Latham E.D., Kassiou M., Werry E.L., Danon J.J. (2024). Emerging TSPO-PET radiotracers for imaging neuroinflammation: a critical analysis. Semin Nucl Med.

[118] Rosenström A.H.C., Konsman J.-P., Kosek E. (2024). Cytokines in cerebrospinal fluid and chronic pain in humans: past, present, and future. Neuroimmunomodulation.

[119] Blichfeldt-Eckhardt M.R., Mortensen W.C.P., Varnum C. (2023). The Danish Pain Research Biobank (DANPAIN-Biobank): a collection of blood, cerebrospinal fluid, and clinical data for the study of neuroimmune and glia-related biomarkers of chronic pain. Ann Transl Med.

[120] Heneka M.T., Rodríguez J.J., Verkhratsky A. (2010). Neuroglia in neurodegeneration. Brain Res Rev.

[121] van der Kant R., Goldstein L.S.B. (2015). Cellular functions of the amyloid precursor protein from development to dementia. Dev Cell.

[122] Sisodia S.S. (1992). Secretion of the beta-amyloid precursor protein. Ann N Y Acad Sci.

[123] Dobrowolska J.A., Michener M.S., Wu G. (2014). CNS amyloid-beta, soluble APP-alpha and -beta kinetics during BACE inhibition. J Neurosci.

[124] Bloom G.S. (2014). Amyloid-β and tau: the trigger and bullet in Alzheimer disease pathogenesis. JAMA Neurol.

[125] Wang Y., Mandelkow E. (2016). Tau in physiology and pathology. Nat Rev Neurosci.

[126] Guo T., Noble W., Hanger D.P. (2017). Roles of tau protein in health and disease. Acta Neuropathol.

[127] Arakhamia T., Lee C.E., Carlomagno Y. (2020). Posttranslational modifications mediate the structural diversity of tauopathy strains. Cell.

[128] DeTure M.A., Dickson D.W. (2019). The neuropathological diagnosis of Alzheimer's disease. Mol Neurodegener.

[129] Scherder E.J., Sergeant J.A., Swaab D.F. (2003). Pain processing in dementia and its relation to neuropathology. Lancet Neurol.

[130] Dugger B.N., Hidalgo J.A., Chiarolanza G. (2013). The distribution of phosphorylated tau in spinal cords of Alzheimer’s disease and non-demented individuals. J Alzheimer’s Dis.

[131] Wang L., Guo Y., Zhu M. (2016). Detection of hyperphosphorylated tau protein and α-synuclein in spinal cord of patients with Alzheimer’s disease. Neuropsychiatr Dis Treat.

[132] Schmidt M.L., Zhukareva V., Perl D.P. (2001). Spinal cord neurofibrillary pathology in Alzheimer disease and Guam Parkinsonism-dementia complex. J Neuropathol Exp Neurol.

[133] Willis W.D., Westlund K.N. (1997). Neuroanatomy of the pain system and of the pathways that modulate pain. J Clin Neurophysiol.

[134] Rüb U., Del Tredici K., Del Turco D., Braak H. (2002). The intralaminar nuclei assigned to the medial pain system and other components of this system are early and progressively affected by the Alzheimer’s disease-related cytoskeletal pathology. J Chem Neuroanat.

[135] Rodriguez-Vieitez E., Saint-Aubert L., Carter S.F. (2016). Diverging longitudinal changes in astrocytosis and amyloid PET in autosomal dominant Alzheimer’s disease. Brain.

[136] Rüb U., Del Tredici K., Schultz C., Thal D., Braak E., Braak H. (2001). The autonomic higher order processing nuclei of the lower brain stem are among the early targets of the Alzheimer’s disease-related cytoskeletal pathology. Acta Neuropathol.

[137] Andrés-Benito P., Moreno J., Aso E., Povedano M., Ferrer I. (2017). Amyotrophic lateral sclerosis, gene deregulation in the anterior horn of the spinal cord and frontal cortex area 8: implications in frontotemporal lobar degeneration. Aging.

[138] Cao S., Fisher D.W., Yu T., Dong H. (2019). The link between chronic pain and Alzheimer’s disease. J Neuroinflammation.

[139] Kelly S.C., He B., Perez S.E., Ginsberg S.D., Mufson E.J., Counts S.E. (2017). Locus coeruleus cellular and molecular pathology during the progression of Alzheimer’s disease. Acta Neuropathol Commun.

[140] Verkhratsky A., Butt A., Li B. (2023). Astrocytes in human central nervous system diseases: a frontier for new therapies. Signal Transduct Target Ther.

[141] Verkhratsky A., Olabarria M., Noristani H.N., Yeh C.-Y., Rodriguez J.J. (2010). Astrocytes in Alzheimer's disease. Neurotherapeutics.

[142] Wyss-Coray T., Loike J.D., Brionne T.C. (2003). Adult mouse astrocytes degrade amyloid-β in vitro and in situ. Nat Med.

[143] Koistinaho M., Lin S., Wu X. (2004). Apolipoprotein E promotes astrocyte colocalization and degradation of deposited amyloid-β peptides. Nat Med.

[144] Sidoryk-Wegrzynowicz M., Wegrzynowicz M., Lee E., Bowman A.B., Aschner M. (2011). Role of astrocytes in brain function and disease. Toxicol Pathol.

[145] Chun H., Kim Y., Kang Y.J. (2020). Severe reactive astrocytes precipitate pathological hallmarks of Alzheimer's disease via excessive H2O2-production. Nat Neurosci.

[146] Zhang Y., Sloan S.A., Clarke L.E. (2016). Purification and characterization of progenitor and mature human astrocytes reveals transcriptional and functional differences with mouse. Neuron.

[147] Malm T.M., Jay T.R., Landreth G.E. (2015). The evolving biology of microglia in Alzheimer's disease. Neurotherapeutics.

[148] van Olst L., Simonton B., Edwards A.J. (2025). Microglial mechanisms drive amyloid-β clearance in immunized patients with Alzheimer’s disease. Nat Med.

[149] Heneka M.T., Golenbock D.T., Latz E. (2015). Innate immunity in Alzheimer's disease. Nat Immunol.

[150] Butovsky O., Weiner H.L. (2018). Microglial signatures and their role in health and disease. Nat Rev Neurosci.

[151] Lee S., Xu G., Jay T.R. (2014). Opposing effects of membrane-anchored CX3CL1 on amyloid and tau pathologies via the p38 MAPK pathway. J Neurosci.

[152] Aman Y., Pitcher T., Simeoli R., Ballard C., Malcangio M. (2016). Reduced thermal sensitivity and increased opioidergic tone in the TASTPM mouse model of Alzheimer’s disease. Pain.

[153] Pugh P.L., Richardson J.C., Bate S.T., Upton N., Sunter D. (2007). Non-cognitive behaviours in an APP/PS1 transgenic model of Alzheimer's disease. Behav Brain Res.

[154] Aman Y., Pitcher T., Ballard C., Malcangio M. (2019). Impaired chronic pain-like behaviour and altered opioidergic system in the TASTPM mouse model of Alzheimer’s disease. Eur J Pain.

[155] Silva R., Sideris-Lampretsas G., Fox S., Zeboudj L., Malcangio M. (2022). CD206+/MHCII− macrophage accumulation at nerve injury site correlates with attenuation of allodynia in TASTPM mouse model of Alzheimer’s disease. Brain Behav Immun Health.

[156] Shukla M., Quirion R., Ma W. (2013). Reduced expression of pain mediators and pain sensitivity in amyloid precursor protein over-expressing CRND8 transgenic mice. Neuroscience.

[157] Mellone M., Kestoras D., Andrews M.R. (2013). Tau pathology is present in vivo and develops in vitro in sensory neurons from human P301S tau transgenic mice: a system for screening drugs against tauopathies. J Neurosci.

[158] López-González I., Schlüter A., Aso E. (2015). Neuroinflammatory signals in Alzheimer disease and APP/PS1 transgenic mice. J Neuropathol Exp Neurol.

[159] Takeuchi H., Iba M., Inoue H. (2011). P301S Mutant human tau transgenic mice manifest early symptoms of human tauopathies with dementia and altered sensorimotor gating. PLoS One.

[160] Oddo S., Caccamo A., Shepherd J.D. (2003). Triple-transgenic model of Alzheimer's disease with plaques and tangles: intracellular Aβ and synaptic dysfunction. Neuron.

[161] Baeta-Corral R., Defrin R., Pick C.G., Giménez-Llort L. (2015). Tail-flick test response in 3×Tg-AD mice at early and advanced stages of disease. Neurosci Lett.

[162] Bhaskar K., Konerth M., Kokiko-Cochran O.N., Cardona A., Ransohoff R.M., Lamb B.T. (2010). Regulation of tau pathology by the microglial fractalkine receptor. Neuron.

[163] Colonna M., Wang Y. (2016). TREM2 variants: new keys to decipher Alzheimer disease pathogenesis. Nat Rev Neurosci.

[164] Shi Y., Holtzman D.M. (2018). Interplay between innate immunity and Alzheimer disease: APOE and TREM2 in the spotlight. Nat Rev Immunol.

[165] Wang X., Zhang S., Lin F., Chu W., Yue S. (2015). Elevated galectin-3 levels in the serum of patients with Alzheimer's disease. Am J Alzheimers Dis Other Demen.

[166] Ulrich J.D., Finn M.B., Wang Y. (2014). Altered microglial response to Aβ plaques in APPPS1-21 mice heterozygous for TREM2. Mol Neurodegener.

[167] Song W.M., Joshita S., Zhou Y., Ulland T.K., Gilfillan S., Colonna M. (2018). Humanized TREM2 mice reveal microglia-intrinsic and -extrinsic effects of R47H polymorphism. J Exp Med.

[168] Parhizkar S., Arzberger T., Brendel M. (2019). Loss of TREM2 function increases amyloid seeding but reduces plaque-associated ApoE. Nat Neurosci.

[169] Leyns C.E.G., Ulrich J.D., Finn M.B. (2017). TREM2 deficiency attenuates neuroinflammation and protects against neurodegeneration in a mouse model of tauopathy. Proc Natl Acad Sci USA.

[170] Mathys H., Davila-Velderrain J., Peng Z. (2019). Single-cell transcriptomic analysis of Alzheimer’s disease. Nature.

[171] Gerrits E., Brouwer N., Kooistra S.M. (2021). Distinct amyloid-β and tau-associated microglia profiles in Alzheimer’s disease. Acta Neuropathol.

[172] Hong S., Beja-Glasser V.F., Nfonoyim B.M. (2016). Complement and microglia mediate early synapse loss in Alzheimer mouse models. Science.

[173] Palop J.J., Mucke L. (2016). Network abnormalities and interneuron dysfunction in Alzheimer disease. Nat Rev Neurosci.

[174] Sadlon A., Takousis P., Ankli B., Alexopoulos P., Perneczky R. (2024). Association of chronic pain with biomarkers of neurodegeneration, microglial activation, and inflammation in cerebrospinal fluid and impaired cognitive function. Ann Neurol.

[175] Scherder E., Oosterman J., Swaab D. (2005). Recent developments in pain in dementia. BMJ.

[176] Jensen-Dahm C., Gasse C., Astrup A., Mortensen P.B., Waldemar G. (2015). Frequent use of opioids in patients with dementia and nursing home residents: a study of the entire elderly population of Denmark. Alzheimers Dement.

[177] Lövheim H., Karlsson S., Gustafson Y. (2008). The use of central nervous system drugs and analgesics among very old people with and without dementia. Pharmacoepidemiol Drug Saf.

[178] Morrison R.S., Siu A.L. (2000). A comparison of pain and its treatment in advanced dementia and cognitively intact patients with hip fracture. J Pain Symptom Manage.

[179] Feldt K.S., Ryden M.B., Miles S. (1998). Treatment of pain in cognitively impaired compared with cognitively intact older patients with hip-fracture. J Am Geriatr Soc.

[180] Erdal A., Ballard C., Vahia I.V., Husebo B.S. (2019). Analgesic treatments in people with dementia - how safe are they? A systematic review. Expert Opin Drug Saf.

[181] Lukas A., Hagg-Grün U., Mayer B., Fischer T., Schuler M. (2019). Pain assessment in advanced dementia. Pain.

[182] Manfredi P.L., Breuer B., Wallenstein S., Stegmann M., Bottomley G., Libow L. (2003). Opioid treatment for agitation in patients with advanced dementia. Int J Geriatr Psychiatry.

[183] Husebo B.S., Ballard C., Sandvik R., Nilsen O.B., Aarsland D. (2011). Efficacy of treating pain to reduce behavioural disturbances in residents of nursing homes with dementia: cluster randomised clinical trial. BMJ.

[184] Erdal A., Flo E., Aarsland D., Ballard C., Slettebo D.D., Husebo B.S. (2018). Efficacy and safety of analgesic treatment for depression in people with advanced dementia: randomised, multicentre, double-blind, placebo-controlled trial (DEP.PAIN.DEM). Drugs Aging.

[185] Oh T.K., Song I.-A. (2024). Impact of prescribed opioid use on development of dementia among patients with chronic non-cancer pain. Sci Rep.

[186] Hukins D., Macleod U., Boland J.W. (2019). Identifying potentially inappropriate prescribing in older people with dementia: a systematic review. Eur J Clin Pharmacol.

[187] Atri A. (2011). Effective pharmacological management of Alzheimer's disease. Am J Manag Care.

[188] Esquerda-Canals G., Montoliu-Gaya L., Güell-Bosch J., Villegas S. (2017). Mouse models of Alzheimer’s disease. J Alzheimers Dis.

[189] Sasaguri H., Hashimoto S., Watamura N. (2022). Recent advances in the modeling of Alzheimer's disease. Front Neurosci.

[190] Howlett D.R., Richardson J.C., Austin A. (2004). Cognitive correlates of Aβ deposition in male and female mice bearing amyloid precursor protein and presenilin-1 mutant transgenes. Brain Res.

[191] Taub D.G., Jiang Q., Pietrafesa F. (2024). The secondary somatosensory cortex gates mechanical and heat sensitivity. Nat Commun.

[192] Muhlbauer M., Metcalf J.C., Robertson J.T., Fridland G., Desiderio D.M. (1986). Opioid peptides in the cerebrospinal fluid of Alzheimer patients. Biomed Chromatogr.

[193] Chishti M.A., Yang D.-S., Janus C. (2001). Early-onset amyloid deposition and cognitive deficits in transgenic mice expressing a double mutant form of amyloid precursor protein 695. J Biol Chem.

[194] Sakakibara Y., Sekiya M., Saito T., Saido T.C., Iijima K.M. (2018). Cognitive and emotional alterations in App knock-in mouse models of Aβ amyloidosis. BMC Neurosci.

[195] Yoshiyama Y., Higuchi M., Zhang B. (2007). Synapse loss and microglial activation precede tangles in a P301S tauopathy mouse model. Neuron.

[196] Sotiropoulos I., Lopes A.T., Pinto V. (2014). Selective impact of Tau loss on nociceptive primary afferents and pain sensation. Exp Neurol.

[197] Boza-Serrano A., Vrillon A., Minta K. (2022). Galectin-3 is elevated in CSF and is associated with Aβ deposits and tau aggregates in brain tissue in Alzheimer’s disease. Acta Neuropathol.

[198] Ashraf G.M., Baeesa S.S. (2018). Investigation of Gal-3 expression pattern in serum and cerebrospinal fluid of patients suffering from neurodegenerative disorders. Front Neurosci.

[199] Yazar T., Olgun Yazar H., Cihan M. (2021). Evaluation of serum galectin-3 levels at Alzheimer patients by stages: a preliminary report. Acta Neurol Belg.

[200] Krasemann S., Madore C., Cialic R. (2017). The TREM2-APOE pathway drives the transcriptional phenotype of dysfunctional microglia in neurodegenerative diseases. Immunity.

[201] Safaiyan S., Besson-Girard S., Kaya T. (2021). White matter aging drives microglial diversity. Neuron.

[202] Holtman I.R., Raj D.D., Miller J.A. (2015). Induction of a common microglia gene expression signature by aging and neurodegenerative conditions: a co-expression meta-analysis. Acta Neuropathol Commun.

[203] Boza-Serrano A., Ruiz R., Sanchez-Varo R. (2019). Galectin-3, a novel endogenous TREM2 ligand, detrimentally regulates inflammatory response in Alzheimer’s disease. Acta Neuropathol.

[204] Johnson E.C.B., Dammer E.B., Duong D.M. (2020). Large-scale proteomic analysis of Alzheimer’s disease brain and cerebrospinal fluid reveals early changes in energy metabolism associated with microglia and astrocyte activation. Nat Med.

[205] Tao C.-C., Cheng K.-M., Ma Y.-L. (2020). Galectin-3 promotes Aβ oligomerization and Aβ toxicity in a mouse model of Alzheimer’s disease. Cell Death Differ.

[206] Siew J.J., Chen H.-M., Chiu F.-L. (2024). Galectin-3 aggravates microglial activation and tau transmission in tauopathy. J Clin Invest.

[207] Burguillos M.A., Svensson M., Schulte T. (2015). Microglia-secreted galectin-3 acts as a toll-like receptor 4 ligand and contributes to microglial activation. Cell Rep.

[208] Shan L., Xu K., Ji L. (2024). Injured sensory neurons-derived galectin-3 contributes to neuropathic pain via programming microglia in the spinal dorsal horn. Brain Behav Immun.

[209] Takasaki I., Taniguchi K., Komatsu F. (2012). Contribution of spinal galectin-3 to acute herpetic allodynia in mice. Pain.

[210] Ma Z., Han Q., Wang X., Ai Z., Zheng Y. (2016). Galectin-3 inhibition is associated with neuropathic pain attenuation after peripheral nerve injury. PLoS One.

[211] Meijers W.C., van der Velde A.R., Pascual-Figal D.A., de Boer R.A. (2015). Galectin-3 and post-myocardial infarction cardiac remodeling. Eur J Pharmacol.

[212] Song L., Tang J., wu, Owusu L., Sun M.-Z., Wu J., Zhang J. (2014). Galectin-3 in cancer. Clinica Chimica Acta.

[213] Rahimian R., Beland L.C., Sato S., Kriz J. (2021). Microglia-derived galectin-3 in neuroinflammation; a bittersweet ligand?. Med Res Rev.

[214] Bouffette S., Botez I., De Ceuninck F. (2023). Targeting galectin-3 in inflammatory and fibrotic diseases. Trends Pharmacol Sci.

[215] Yu X., Basbaum A., Guan Z. (2021). Contribution of colony-stimulating factor 1 to neuropathic pain. Pain Rep.

[216] Gonçalves Dos Santos G., Delay L., Yaksh T.L., Corr M. (2020). Neuraxial cytokines in pain states. Front Immunol.

[217] Pons V., Rivest S. (2022). Targeting systemic innate immune cells as a therapeutic avenue for Alzheimer disease. Pharmacol Rev.

[218] Majumdar A., Cruz D., Asamoah N. (2007). Activation of microglia acidifies lysosomes and leads to degradation of Alzheimer amyloid fibrils. Mol Biol Cell.

[219] Sassi C., Nalls M.A., Ridge P.G. (2018). Mendelian adult-onset leukodystrophy genes in Alzheimer's disease: critical influence of CSF1R and NOTCH3. Neurobiol Aging.

[220] Olmos-Alonso A., Schetters S.T.T., Sri S. (2016). Pharmacological targeting of CSF1R inhibits microglial proliferation and prevents the progression of Alzheimer's-like pathology. Brain.

[221] Mancuso R., Fryatt G., Cleal M. (2019). CSF1R inhibitor JNJ-40346527 attenuates microglial proliferation and neurodegeneration in P301S mice. Brain.

[222] Heneka M.T. (2017). Inflammasome activation and innate immunity in Alzheimer's disease. Brain Pathol.

[223] O’Bryant S.E., Waring S.C., Cullum C.M. (2008). Staging dementia using Clinical Dementia Rating Scale Sum of Boxes scores: a Texas Alzheimer’s research consortium study. Arch Neurol.

